# Drug-Metabolizing Enzymes in Human Keratinocytes and In Vitro Detection of Cytochrome P450-Mediated Phenolic Lamotrigine Metabolite

**DOI:** 10.1021/acs.chemrestox.5c00500

**Published:** 2026-03-30

**Authors:** Philipp N. Deck, Mareike Müller, Andreas Glässner, Matthias Vogel, Michael Steffens, Caspar Heubach, Guido Fechner, Karl Becker, Günther Weindl, Bernhardt Sachs

**Affiliations:** † Research Division, 9195 Federal Institute for Drugs and Medical Devices (BfArM), Bonn 53175, Germany; ‡ Pharmacopoeia and Standard Licensing Division, Federal Institute for Drugs and Medical Devices (BfArM), Bonn 53175, Germany; § Department of Urology and Pediatric Urology, 39062 University Hospital Bonn, Bonn 53127, Germany; ∥ Kinderchirurgische Gemeinschaftspraxis, Bonn 53113, Germany; ⊥ Pharmacology and Toxicology, Pharmaceutical Institute, University of Bonn, Bonn 53121, Germany

## Abstract

Drug-induced hypersensitivity reactions can manifest as severe cutaneous adverse reactions, with lamotrigine (LTG) being a known elicitor in a subset of patients. While reactive metabolites are proposed to play a role, the contribution of skin metabolism is not well established. Here, we characterized drug-metabolizing enzymes and transporters in primary human keratinocytes and assessed the LTG metabolism in vitro. Using a combined transcriptomic and metabolomic approach, we demonstrated that primary human keratinocytes show a limited drug-metabolizing capacity. Although xenobiotic-metabolizing enzymes and drug transporters (phases I-III) were expressed at the transcript level, key hepatic cytochrome P450 enzymes (CYPs) were undetectable at both mRNA and protein levels. Following LTG incubation, low levels of LTG-N2-glucuronide were formed in keratinocytes as well as (reactive) metabolites, including a glutathione (GSH) adduct and a putative phenolic LTG derivative, in several in vitro assays. LTG-N2-sulfate formation could not be achieved under aqueous conditions. Transcriptome profiling of keratinocytes revealed no significant response to LTG, LTG-N2-oxide, or LTG+valproate, whereas interferon-γ triggered a pronounced proinflammatory gene expression signature. These findings provide new data of LTG metabolism, highlighting a novel CYP-derived phenolic pathway linked to GSH conjugation.

## Graphical abstract

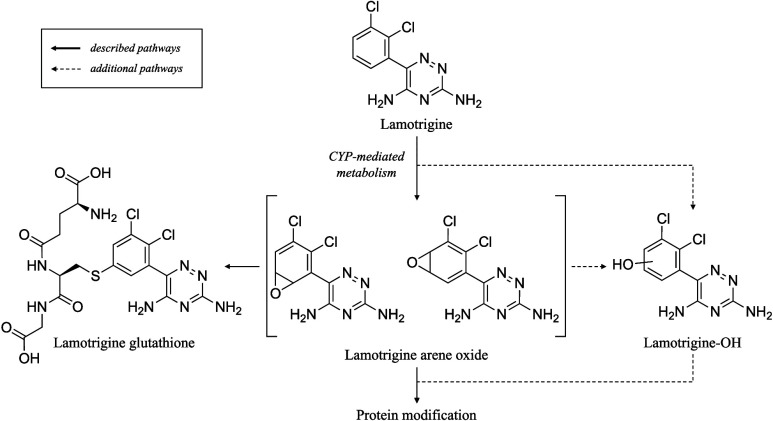

## Introduction

In recent decades, the skin, particularly keratinocytes, has been recognized as an extrahepatic metabolic site capable of converting both endogenous and exogenous substances.^1^ The enzymatic profile is primarily characterized by phase II enzymes, while phase I enzymes such as cytochrome P450 enzymes (CYPs) are less common and different isoforms are expressed compared to the liver.^1–5^ The skin may thus also play a potential role in drug metabolism, particularly in the case of adverse drug reactions (ADRs).^6^

Cutaneous drug hypersensitivity reactions (cDHRs) represent a heterogeneous group of ADRs, ranging from mild skin eruptions to life-threatening severe cutaneous adverse reactions (SCARs), such as Stevens-Johnson syndrome and toxic epidermal necrolysis.^7^ The underlying mechanisms of cDHRs are still not yet fully elucidated.^8^ One hypothesis points to the role of chemically reactive drug metabolites in triggering immune responses. These reactive metabolites are typically unstable and short-lived, acting locally at the site of formation. They may modify endogenous proteins and create neoantigens, or directly induce cellular stress, both of which can lead to T-cell activation.^9^ A further hypothesized mechanism proposes that the drug itself may directly interact with the T-cell receptor, thereby contributing to T-cell activation without prior metabolic transformation or antigen processing leading to interferon-γ (IFN-γ) secretion.^10^ Genetic predisposition also plays a role, particularly through alleles of the human leukocyte antigen (HLA). For example, HLA-B*58:01 is associated with lamotrigine-induced SCARs in Han Chinese.^11^

Lamotrigine (LTG), widely prescribed as an antiepileptic, is generally well tolerated; however, skin reactions occur in up to 10% of patients. SCARs, although less common than mild cutaneous reactions, occur in approximately 0.3% of patients, a considerable rate given the severity of these reactions.^12,13^ For LTG, hepatic and extrahepatic bioactivation pathways have been proposed, leading to the formation of reactive intermediates such as arene oxides.^14,15^ Furthermore, LTG-N2-oxide, a phase I metabolite, appears to be a substrate for human sulfotransferases and could be converted into a chemically reactive LTG-N2-sulfate.^16^ There are also suggestions that LTG itself may be responsible for the cutaneous ADRs observed in some patients (see Scheme 1).^10,17^

**Scheme 1:**
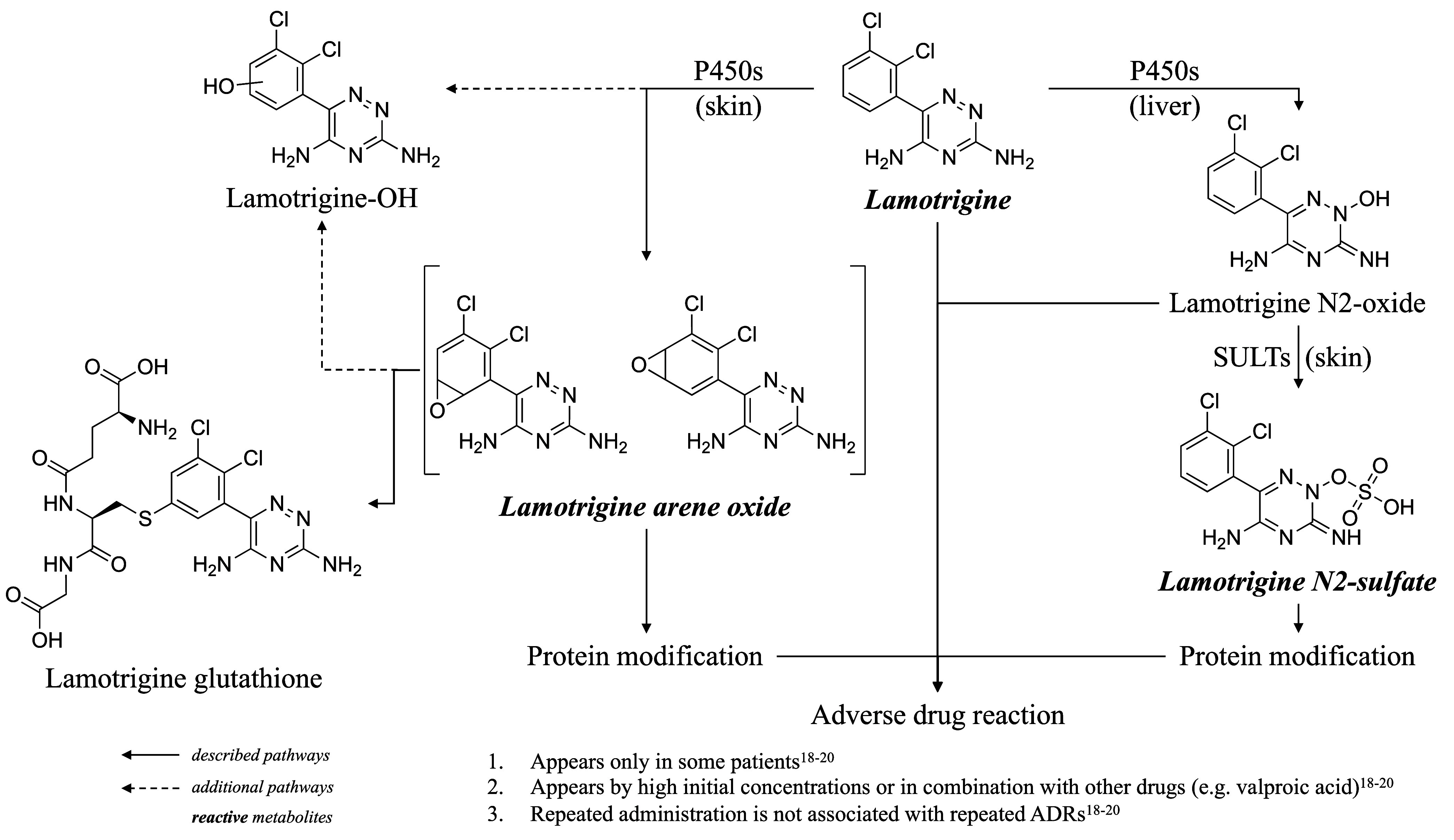
Visualization of Hypothesized Metabolic Pathways of LTG and Its (Reactive) Metabolites That Could Lead to an Adverse Drug Reaction, Including Potential Additional Pathways Identified in This Study^a^ ^,^ ^18–20^

a There are currently multiple hypotheses that attempt to explain the LTG-induced hypersensitivity reactions via different pathways. Two chemically reactive metabolites are considered as possible triggers: LTG arene oxide, which is thought to be formed by CYP-mediated oxidation in the skin,^14,15^ and LTG-N2-sulfate, which is assumed to be formed after hepatic formation of LTG-N2-oxide and subsequent sulfation in the skin.^16^ Additionally, a direct pharmacological interaction (p-i concept) of LTG with immune receptors is discussed.^10,21^

The metabolism of LTG has been extensively studied;^22^ however, the mechanisms by which LTG may trigger cDHRs remain under debate.^7,16,23^ In this study, we used different experimental approaches (see Scheme S1) to reproduce previous key findings, study proposed metabolic pathways, and identify inconsistencies or gaps in the current understanding.

## Materials and Methods

### Isolation and Stimulation of Primary Human Keratinocytes

Primary human keratinocytes were isolated from excess skin tissue obtained during routine circumcision (foreskin) and plastic surgery (abdominal skin) using a slightly modified procedure as described previously.^24^ Briefly, skin samples were first immersed in phosphate-buffered saline (PBS; L0615-500, Biowest, Nuaillé, France) for 2 min and then washed in Dermacult (100-0500, Stemcell Technologies Saint Égrève, France), including additives penicillin/streptomycin (L0022-100, Biowest) and ampothericin B (1 μg/mL; 15290026, Life Technologies GmbH, Darmstadt, Germany). The tissue was cut into 1 × 1 cm pieces and incubated overnight at 4 °C in Dispase II solution (04942078001, Sigma-Aldrich Chemie GmbH, Taufkirchen, Germany). On the following day, the epidermis was removed from the underlying dermis and incubated for a maximum of 30 min at 37 °C in prewarmed TrypLE Express (12604-013, Life Technologies GmbH). Enzymatic digestion was stopped by adding 7 mL of PBS, and the cell suspension was passed through a 100 μm cell strainer (83.3945.100, Sarstedt, Nümbrecht, Germany). The eluate was centrifuged at 0.2 rcf for 10 min. Cells were resuspended in 1 mL of Dermacult and seeded in two T75 flasks containing 10 mL of Dermacult. In addition, part of the epidermal tissue was reserved for the isolation of RNA and proteins prior to enzymatic digestion.

To investigate metabolic activity in keratinocytes, calcium-differentiated (1.2 mM) keratinocytes were incubated with LTG for 24 h. The concentration was adjusted depending on the metabolite to be investigated. Concentrations of 0, 1, 10, and 100 μM were selected for the detection of the two main metabolites, LTG-N2-glucuronide and LTG-N2-oxide. For the detection of LTG-GSH, 500 μM was used as described.^15^ The cells were then washed with PBS, centrifuged at 0.6 rcf for 5 min, and the supernatant and pellet were frozen (−20 °C) until further processing.

### Human and Rat Subcellular Liver Fractions Assays

To investigate the metabolism of LTG, in vitro incubation assays were performed using human and rat subcellular liver fractions. The incubations were carried out in 1.5 mL microcentrifuge tubes at 37 °C in a thermal shaker for 2 h at 600 rpm. The final mixture (total volume: 200 μL) contained 1 mg/mL subcellular fraction (either hS9, HLM, HLC, RLM (HMS9PL; HMMCPL; HMCYPL; RTMCPL), Life Technologies GmbH; or rS9, ICCR-Roßdorf GmbH, Roßdorf, Germany), 10 mM phosphate buffer (pH 7.5), 1 mM magnesium chloride (M8266, Sigma-Aldrich Chemie GmbH), 1 mM NADPH (16156, Biomol, Hamburg, Germany), and, depending on the assay, either 1 mM reduced glutathione (GSH; G4251, Sigma-Aldrich Chemie GmbH), uridine-5′-diphosphoglucuronic acid (UDPGA; U6751, Sigma-Aldrich Chemie GmbH) or adenosine 3′-phosphate 5′-phosphosulfate triethylammonium salt (PAPS; 94455, Sigma-Aldrich Chemie GmbH). LTG was used at a final concentration of 2.5 μM unless otherwise specified. NADPH was added after a preincubation of 5 min. The reactions were terminated by adding 600 μL of acetonitrile (ACN). Samples were vortexed and centrifuged at 16,000 *g* for 5 min at 4 °C, and the supernatants were collected for liquid chromatography-mass spectrometry (LC-MS) analysis. Negative controls (substrate blank, enzyme blank) and positive controls (acetaminophen, 2,5 μM; Cay10024, Biomol, Germany) were included. All incubations were performed in at least in triplicate.

### Recombinant CYP Enzyme Assays

Analogous to subcellular fractions, incubation experiments were carried out with five recombinant cytochrome P450 enzymes (rCYPs), whereby the rCYP concentration was 20 pgmol/ml and the LTG concentration was 500 μM. The following CYPs were examined: rCYP2A6 (CYP/EZ011, Cypex Ltd., Scotland, UK), rCYP2B6 (CYP/EZ020, Cypex Ltd.), rCYP2D6 (CYP/EZ007, Cypex Ltd.), rCYP2E1 (CYP/EZ009, Cypex Ltd.), and rCYP3A4 (CYP/EZ002, Cypex Ltd.). Coumarin (1 μM, CYP2A6; C4261, Sigma-Aldrich Chemie GmbH), bupropion (2.5 μM, CYP2B6; J61105, Life Technologies GmbH), dextromethorphan (2.5 μM, CYP2D6; EPD0740000, LGC Standards), chlorzoxazone (2.5 μM, CYP2E1; C4397, Sigma-Aldrich Chemie GmbH), and testosterone (0.5 μM, CYP3A4; 86500, Sigma-Aldrich Chemie GmbH) were used as positive controls for enzyme turnover, and acetaminophen was again used as a positive control for the formation of a GSH adduct. In addition, we investigated the formation of GSH adducts using LTG-d_3_ (Cay37196, Biomol) in incubations with rCYP2D6.

### Synthesis of LTG Metabolites

The synthesis of LTG-N2-oxide and the potentially reactive metabolite LTG-N2-sulfate was carried out according to the procedure described previously.^16^ The purification of LTG-N2-sulfate was attempted using a Knauer high-performance liquid chromatograph (HPLC) (AZURA Autosampler AS 6.1L, AZURA P 6.1L HPLC Pump) equipped with a UV-DAD detector (AZURA DAD 2.1L). Additional approaches included solid-phase extraction (SPE, CHROMABOND C18, 1 mL/100 mg, MACHEREY-NAGEL) and thin-layer chromatography (TLC). The TLC analysis of the synthesized LTG-N2-sulfate was performed using CHCl_3_/MeOH/H_2_O/AcOH (65:25:5:1) as the mobile phase and rhodamine B (CP37.1, Carl Roth GmbH + Co. KG) as the visualization indicator. Further details on the TLC plate and identification of LTG-N2-sulfate by LC-MS without any purification can be found in the supplement (see Figures S1 and S2).

### Isolation of RNA and Protein

Total RNA was extracted from keratinocytes exposed to the conditions outlined elsewhere using the RNeasy Mini Kit (74104, QIAGEN GmbH) following the manufacturer’s instructions. RNA concentration and purity were assessed using a Nanodrop 1000 spectrophotometer. Samples were stored at −80 °C until further processing.

Protein isolation was performed using RIPA buffer containing a mixture of phosphatase (4906845001, Sigma-Aldrich Chemie GmbH) and protease inhibitors (4693124001, Sigma-Aldrich Chemie GmbH), and total protein concentration was determined using Pierce’s BCA protein assay kit (23225, Life Technologies GmbH) according to the manufacturer’s instructions.

### Transcriptomics

For RNA sequencing analyses, 5 × 10^5^ cells per 6 cm dish were seeded and exposed to one of the following conditions: LTG (600 μM; PHR1392, Sigma-Aldrich Chemie GmbH), LTG-N2-oxide (500 μM; self-synthesized), LTG with valproic acid (600 μM/100 μM; P4543, Sigma-Aldrich Chemie GmbH), LTG with IFN-γ (600 μM/100 μg/mL; 11343534, ImmunoTools GmbH, Friesoythe, Germany), IFN-γ alone (100 μg/mL), or medium only. The incubation times were 2 and 6 h. Each condition was performed in biological triplicate using cells from three individual donors. Total RNA isolation was performed as described above. Additional quality control steps, library preparation, and bulk RNA sequencing were conducted by the NGS Core Facility at the University of Bonn, Germany. Library prep was performed using the Lexogen QuantSeq 3′ mRNA-Seq Library Prep Kit FWD and 200 ng of RNA as input. Sequencing was done with 1 × 100 bp on a NovaSeq 6000 S1 100 Flow cell and with 10 M raw reads per sample. The bioinformatic analysis of the sequencing data was performed in-house as previously described.^25^ Briefly, sequencing reads were aligned to the human reference genome GRCh38 (hg38), and differential expression analysis was performed using DESeq2 (Version 1.44.0).

### Quantitative RT-PCR (qPCR)

For qPCR analyses, keratinocytes were seeded at 5 × 10^5^ cells per 6 cm dish and incubated with or without 1.2 mM calcium chloride (C7902, Sigma-Aldrich Chemie GmbH) for 24 h. qPCR was performed using the QuantiTect SYBR Green RT-PCR Kit (204243, QIAGEN GmbH) and SYBR Green PCR Master Mix (204145, QIAGEN GmbH) according to the manufacturer’s instructions. Briefly, the thermal cycling protocol included an initial preincubation step at 95 °C for 10 min, followed by 40 amplification cycles consisting of denaturation at 94 °C for 15 s with a ramp rate of 2.5 °C/s, annealing at 55 °C for 30 s with a ramp rate of 2.2 °C/s, and elongation at 72 °C for 30 s with a ramp rate of 2.2 °C/s. Fluorescence was measured at the end of each extension phase. To confirm the specificity of amplification, a melting curve analysis was performed following the PCR run.

Basal gene expression of 19 target genes for phase I and II drug-metabolizing enzymes was analyzed (*CYP1B1*, *CYP2A6*, *CYP2B6*, *CYP2C8*, *CYP2C9*, *CYP2C19*, *CYP2D6*, *CYP2E1*, *CYP2J2*, *CYP3A4*, *SULT1A1*, *SULT1A2*, *SULT1C4*, *UGT1A1*, *UGT1A4*, *UGT2B7*, *PTGS1*, *PTGS2*, and *COMT*). In addition, the gene expression of *KRT14* and *LOR* was analyzed as a quality control for the current differentiation status of the keratinocytes. Gene expression was normalized to that of *GAPDH*. Results were categorized based on Δ*Ct* values as follows: “+++” (strong expression, Δ*Ct* < 5), “++” (moderate expression, Δ*Ct* 5–10), “+” (low expression, Δ*Ct* > 10), “–” (no expression), or “ND” (not determined). Primer sequences are provided in Supporting Information Table S1.

### Western Blot

For Western blot analyses, keratinocytes were seeded at 5 × 10^5^ cells per 6 cm dish, and HepG2 cells at 1 × 10^6^ cells per 6 cm dish. Cells were incubated either with rifampicin at a concentration of 60.7 μM (50 μg/mL) or without rifampicin (R3501, Sigma-Aldrich Chemie GmbH). To induce keratinocyte differentiation, 1.2 mM calcium chloride was added. Conditions were maintained for either 48 or 96 h. Protein extraction was performed as described above. Furthermore, epidermal tissue obtained from six individual foreskin donors was processed under analogous conditions to generate total cell lysates.

Self-cast stain-free 10% TGX-polyacrylamide gels (1610183, Bio-Rad, Feldkirchen, Germany) were prepared using ammonium persulfate (9592.2, Carl Roth GmbH + Co. KG, Karlsruhe, Germany) and TEMED (2367.3, Carl Roth GmbH + Co. KG) as polymerization initiators. The protein samples were prepared in Laemmli sample buffer (1610747, Bio-Rad) with the addition of dithiothreitol (6908.3, Carl Roth GmbH + Co. KG). Each sample was loaded with 20 μg of total protein except for human liver microsomes (60 μg of protein, Life Technologies GmbH), which served as a positive control. Proteins were first separated by electrophoresis at 100 V for 1.5 h and then transferred with CAPS buffer (pH: 9.6;1610778, Bio-Rad) to microporous PVDF membranes (1620177, Bio-Rad) at 4 °C for 2 h. The membranes were incubated overnight at 4 °C with primary antibodies, followed by a 1 h incubation with horseradish peroxidase (HRP)-conjugated secondary antibodies at room temperature. Protein bands were visualized using a Clarity Max Western ECL substrate (1705062, Bio-Rad). The following antibodies were used: CYP2C8 (1:1000; PA582486, RRID: AB_2789644, Life Technologies GmbH), CYP2C9 (1:1000; PA5120976, RRID: AB_2914548, Life Technologies GmbH), CYP2C19 (1:1000; PA5112395, RRID: AB_2867130, Life Technologies GmbH), CYP2D6 (1:1000; PA535148, RRID: AB_2552458, Life Technologies GmbH), CYP2E1 (1:1000; PA582486, RRID: AB_2552661, Life Technologies GmbH), CYP3A4 (1:1000; PA582486, RRID: AB_2090334, Life Technologies GmbH), GAPDH (1:1000; sc-47724, RRID: AB_637678, clone id: 0411, Santa Cruz Biotechnology, Heidelberg, Germany), anti-rabbit (1:10,000; 31464, RRID: AB_228378, Life Technologies GmbH), anti-mouse (1:10,000; M32407, RRID: AB_10563452, Life Technologies GmbH).

### HPLC-MS

Samples were analyzed using liquid chromatography coupled to mass spectrometry. Chromatographic separation was performed on InfinityLab Poroshell 120 EC-C18, 2.1 × 100 mm, 2.7 μm (695975-302, Agilent Technologies Deutschland GmbH, Waldbronn, Germany) maintained at 30 °C using a Shimadzu Nexera UPLC system. The injection volume of each sample was 10 μL. The mobile phase consisted of water with 5 mM ammonium acetate (pH: 5) as the inorganic phase and acetonitrile (ACN) as the organic phase. The following gradient elution was chosen unless otherwise specified: Starting at 1%, the ACN level was increased to 20% for 20 min, then changed to 50% after 22.5 min and held at 50% for 5 min before the column was re-equilibrated with 1% ACN for a further 5 min. The flow rate was 0.35 mL/min. LC-MS analysis was performed using a SCIEX QTRAP 6500 instrument equipped with a Turbo V ion source in positive ESI mode. The analyses were performed in enhanced product ion (EPI) scan mode, unless described otherwise specified in the supplement. Data acquisition and processing were done using Analyst 1.6.2 software. The identification of the compounds was based on the exact mass, the retention time, and their fragmentation pattern, and, if possible, by comparison with existing standards.

### Ethics Statement

The study was approved by the local ethics committee at the University Hospitals of Aachen (UKA 22-001) and Bonn (UKB 205/22) and the North Rhine State Chamber of Physicians (AekNo 2023231). All tissue donors provided written informed consent.

### Statistical Analysis and Data Preparation

Unless otherwise stated, all experiments were performed in biological triplicates, and data are reported as mean values ± SD. Cells were used up to passage 5. Statistical calculations for bulk RNA Sequencing (Wald test *p*-value and Benjamini-Hochberg (BH) adjusted *p*-value) were determined using DESeq2 (Version 1.44.0). MS diagrams were created using Sciex Analyst (version 1.6.2) and PowerPoint.

## Results

### Expression of Drug-Metabolizing Enzymes in Human Keratinocytes

Previous studies have reported variable expression of drug-metabolizing enzymes in human keratinocytes depending on the culture medium, experimental conditions, or source of keratinocytes.^1,5,26^ We therefore reexamined the basal expression profile in keratinocytes. The following results can be found in detail in the supplementary Excel table “Supplementary_RNA-Seq” (Table S2) and online under the GEO series accession number GSE324594 (see Data Availability Statement).

Among the phase I enzymes, several CYPs exhibited transcript abundance. *CYP1B1* showed the highest normalized gene expression among the drug-metabolizing CYPs, followed by those of *CYP20A1*, *CYP26B1*, *CYP27B1*, and *CYP2S1*. Moderate expression was observed for *CYP2E1*, *CYP2J2*, *CYP2R1*, *CYP7B1*, and *CYP4F11*. Transcripts for key hepatic CYPs, including *CYP3A4*, *CYP2C9*, *CYP2C19*, and *CYP2D6*, were not detected.

In addition, several non-CYP Phase I oxidoreductases showed high expression levels. *ALDH1A3*, *ALDH3A1*, *ALDH4A1*, *ALDH7A1*, and *ALDH9A1* were strongly expressed. Members of the aldo-keto reductase family, particularly *AKR1C1*, *AKR1C2*, and *AKR1B1*, also showed high abundance. Within the flavin-containing monooxygenases (FMOs), only *FMO4* was detectably expressed, although at lower levels. Among the phase II drug-metabolizing enzymes, glutathione S-transferases (GSTs) were dominated by *GSTP1*, which showed the highest normalized gene counts. *GSTO1*, *GSTO2*, *GSTM3*, *GSTA4*, *GSTK1*, and *GSTM4* were also expressed to a significant extent. Sulfotransferases (*SULT2B1*, *SULT1A1*, and *SULT1E1)* showed a moderately normalized expression. UDP-glucuronosyltransferase expression was minimal; only *UGT1A10* exceeded the background levels. Among other conjugating enzymes, *COMT* showed strong expression, while *MAOA* and *CES2* were expressed at moderate levels. Phase III transporter expression was dominated by *ABCC3*, followed by *ABCC1*, *ABCC2*, *ABCC4*, *ABCC5*, *ABCC10*, and *ABCG2*. Additional transporters such as *ABCC11* and *SLC22A1* were detected at lower expression levels.

To confirm the transcriptome analysis for selected CYP genes, we performed qPCR analysis with keratinocytes cultured in the presence or absence of calcium (1.2 mM) to identify differences due to differentiation. As expected, loricrin (LOR), used as a differentiation marker,^27^ showed a strong induction from low (+) to high (+++) expression upon calcium incubation, while KRT14 expression remained consistently high (+++).^28^ Except for *CYP2A6*, *CYP2B6*, *CYP2C19*, and *CYP3A4*, low (+) to strong (+++) basal expression levels were observed for the selected genes previously reported to be expressed in keratinocytes.^1^ Analysis of selected phase II enzymes identified low (+) to moderate (++) basal expression levels. *SULT1A1* showed moderate expression (++) (Table 1). No major differences in expression patterns were observed between calcium-stimulated and unstimulated cells for both the phase I and II enzymes.

**Table 1:** Analysis of the Basal Gene Expression of Phase I and II Enzymes by qPCR in Keratinocytes^a^

	keratinocytes			keratinocytes	
	without Ca^2+^	with Ca^2+^		without Ca^2+^	with Ca^2+^
**phase I enzymes**			**phase II enzymes**		
*CYP1B1*	+++	+++	*SULT1A1*	++	nd
CYP2A6*	–	–	*SULT1A2*	++	nd
CYP2B6*	–	–	*SULT1C4*	–	nd
*CYP2C8*	+	+	*UGT1A1*	+	+
*CYP2C9*	+	+	*UGT1A4*	–	–
*CYP2C19*	–	–	*UGT2B7*	+	+
*CYP2D6*	+	+	PTGS1*	++	++
*CYP2E1*	+	+	PTGS2*	++	+
*CYP2J2*	++	++	*COMT*	++	++
*CYP3A4*	–	–			
**differentiation marker genes**					
*LOR*	+	+++	*KRT14*	+++	+++

a Keratinocytes were incubated with or without calcium (1.2 mM) for 24 h. GAPDH served as housekeeping gene. * Data for CYP2A6, CYP2B6, PTGS1, and PTGS2 are shown as biological duplicates. The relative abundance of mRNA is expressed as: – “not detected”, + “weak gene expression, Δ*Ct* > 10”, + + “moderate expression, Δ*Ct* 5–10”, + ++ “high expression, Δ*Ct* < 5”, and nd “not determined”.

Next, we analyzed the protein expression of six major CYPs, CYP2C8, CYP2C9, CYP2C19, CYP2D6, CYP2E1, and CYP3A4, in keratinocytes. HepG2 cells, which express CYPs at very low levels, were used as a reference,^29^ while human liver microsomes (HLM) containing all CYPs served as positive control (Figure 1). Neither induction with calcium alone nor in combination with the CYP450 inductor rifampicin for 48 or 96 h led to detectable expression of any CYP enzymes in keratinocytes. A very weak band corresponding to CYP2C8 was observed in HepG2 cells, while other CYP isoforms were undetectable.

**Figure 1:**
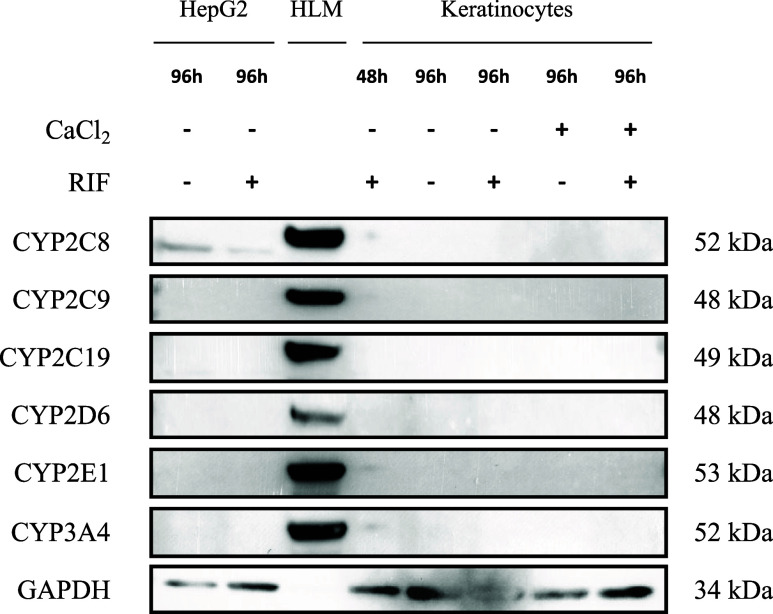
CYP protein levels in HepG2 and keratinocytes with and without rifampicin induction.

This figure shows representative Western blot images of CYP2C8, CYP2C9, CYP2C19, CYP2D6, CYP2E1, and CYP3A4 in HLM, HepG2 cells, and primary keratinocytes. Cells were incubated with or without rifampicin (60.7 μM) and, for keratinocytes, additionally with or without calcium chloride (1.2 mM) for 48 or 96 h. GAPDH was used as a loading control. A total of seven different skin donors were tested for CYP expression.

### Keratinocytes Show No Major Transcriptomic Changes Following Exposure to LTG and LTG-N2-Oxide

Bulk RNA sequencing with differential expression analysis was used to examine transcriptional changes in keratinocytes after exposure to LTG, its CYP-mediated metabolite LTG-N2-oxide, and coincubated LTG with valproic acid (VPA). The rationale for including the latter condition is based on previous reports showing that the addition of LTG to VPA therapy increases the frequency of hypersensitivity reactions.^12,30–32^ IFN-γ served as a positive control, as its effects on keratinocytes are well documented.^33^

Overall, no substantial transcriptional response to LTG, its metabolite, or LTG coincubated with VPA was observed (Table S2). Incubation with LTG led to only two downregulated genes (*SERPINB2*, *CCL20*) detected at 2 hours and none at 6 hours (adjusted *p* < 0.05, |LFC| > 0.58). Exposure to LTG-N2-oxide resulted in the detection of three differentially expressed genes (DEGs) at each time point (2 h: *RPS17*, *GFUS*, *RPS15AP17*; 6 h: *CHAC1*, *RPL5P4*, *HRAS*). Co-incubation with LTG and VPA resulted in two upregulated and four downregulated DEGs identified at 6 h only. Significant changes in gene expression were observed only in keratinocyte cultures stimulated with IFN-γ. After 2 h, a total of 35 DEGs, including 32 upregulated and 3 downregulated genes, were detected. Among the upregulated genes, several chemokine-related transcripts showed a significant induction compared with the unstimulated control. *CXCL9*, *CXCL10*, and *ICAM1* were the most highly upregulated genes, with values of 11.2, 9.1, and 9.5. After 6 h, the transcriptional response to IFN-γ was further enhanced. The expression of *CXCL9*, *CXCL10*, and *ICAM1* remained significantly increased, with further increases in fold-change values (e.g., *CXCL10*: 13.7). In addition, immunoregulatory genes such as *IRF1* and *STAT1* were significantly upregulated (Figure S4). A total of 198 DEGs were detected at this later time point, including 158 upregulated and 40 downregulated genes.

### Detection of LTG Metabolites in Keratinocytes with Evidence for LTG-N2-Glucuronide and LTG-N2-Oxide Formation

To confirm previously described metabolic pathways^22^ of LTG, in vitro incubations of LTG with human keratinocytes and subcellular fractions were performed. Metabolites were identified by using LC-MS (Table 2).

**Table 2:** Summary of the Metabolites Identified in Keratinocytes and Enzyme Assays^a^

metabolite	keratinocytes	subcellular fractions (hS9, HLM,...)
lamotrigine-N2-glucuronide	(+)	+
lamotrigine-N2-oxide	(+)	+
lamotrigine-N2-sulfate	nd	–
lamotrigine-glutathione	–	+

a + “present”, – “absent”, nd “not determined”, () “ambiguous”.

Almost all previously reported metabolites were qualitatively detected in both rat and human subcellular incubation assays (Figures S5–S8). The only exception was LTG-N2-sulfate, which was not observed in any of the assays. In addition, evidence of the formation of LTG-N2-glucuronide (Figure S6) and LTG-N2-oxide was found in calcium-differentiated keratinocyte. We did not observe any formation of LTG-GSH in keratinocytes even after exposure to 500 μM LTG, in contrast to previous reports describing their formation.^15^

Attempts to synthesize LTG-N2-sulfate by reacting LTG-N2-oxide with sulfur trioxide-pyridine in anhydrous DMF, following the reported procedure,^16^ resulted in a product detectable by LC-MS and TLC (Figures S1 and S2). Further purification under aqueous conditions was not possible, presumably due to high instability in water, which could also explain why we were unable to detect LTG-N2-sulfate in an in vitro assay using subcellular fractions.

### Discovery of an Aromatic Hydroxylated Isomer of LTG-N2-Oxide in Subcellular Fractions and Its Enzyme-Dependent Formation

LC-MS analysis revealed a metabolite with an identical precursor ion *m*/*z*-ratio (272.0, [M + H]^+^) to that of LTG-N2-oxide, but with a distinct chromatographic retention time and an altered tandem mass spectrometric (MS/MS) fragmentation profile. Repeat analyses using high-resolution mass spectrometry confirmed the results initially obtained (Figure S9).

In both compounds, the full-scan MS spectra displayed the expected chlorine isotope pattern, which is consistent with the presence of two chlorine atoms. However, the MS/MS analysis revealed distinct fragmentation profiles. The known LTG-N2-oxide exhibited transitions from *m*/*z* 272 to major product ions at *m*/*z* 242, 185, and 165. In contrast, the constitutional isomer of LTG-N2-oxide, herein termed LTG-OH, showed product ions at *m*/*z* 236, 227, 188, and 181 (Figure 2).

**Figure 2:**
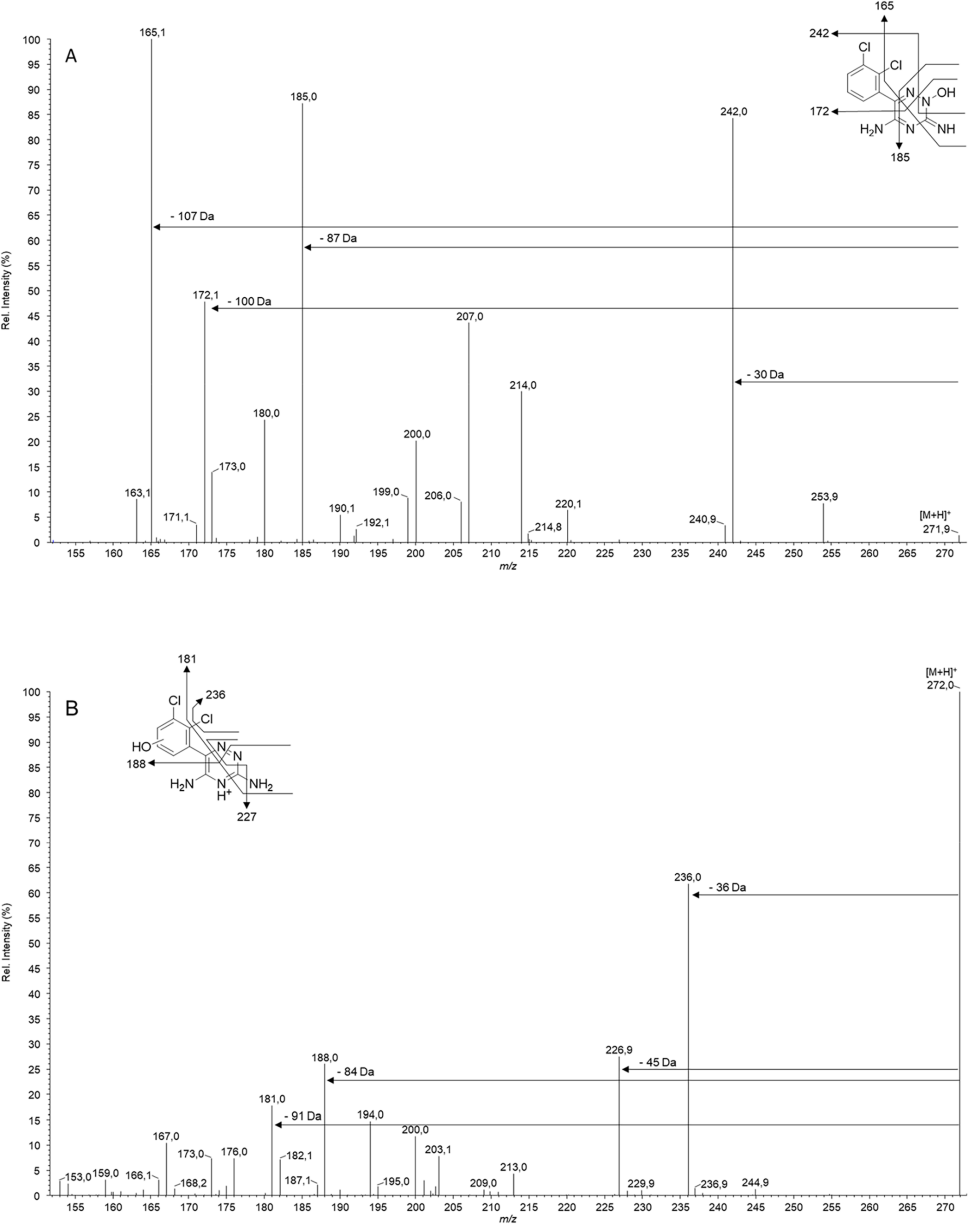
Formation of constitutional isomer of LTG N-2-oxide in subcellular fractions. MS/MS fragmentation spectra of LTG-N2-oxide (A) and its structural isomer LTG-OH (B).

Incubation of LTG with recombinant human CYP2A6, CYP2B6, CYP2D6, CYP2E1, and CYP3A4 in the presence of GSH led to the formation of a GSH adduct and again two constitutional isomers with the same precursor ion mass (*m*/*z* 272.0 [M + H]^+^), analogous to the incubations with the S9 mix. The metabolites formed had identical retention times and fragments as described above. Which constitutional isomer was formed appeared to be also enzyme-dependent, with rCYP2D6 favoring LTG-OH, while rCYP3A4 predominantly produced LTG-N2-oxide. rCYP2A6 likewise appears to prefer the formation of LTG-N2-oxide, while no clear trend was observed for rCYP2B6 and rCYP2E1 (Figures S10–S14).

To further confirm the formation of an aromatic glutathione adduct and the constitutional isomer, rCYP2D6 was incubated with LTG-d_3_ instead of LTG. Adduct formation with GSH and the aromatic hydroxylation were again observed (Figure S15).

## Discussion

### Drug-Metabolizing Enzyme Expression in Keratinocytes Suggests Metabolic Activity for LTG

The skin is a metabolically active organ that can contribute to local drug metabolism.^1^ Since the skin consists largely of keratinocytes, studying their drug-metabolizing expression pattern is essential to understanding how drugs such as LTG can trigger cDHRs. We therefore investigated the expression profile of drug-metabolizing enzymes in human keratinocytes and evaluated potential LTG-associated changes.

In line with previous findings,^2,3,26,34^ our results confirm that keratinocytes express a variety of phase I and phase II drug-metabolizing enzymes as well as phase III transporters. Several of these enzymes have been previously associated with LTG metabolism,^22^ suggesting that keratinocytes may indeed be capable of metabolizing LTG.

As for phase I enzymes, CYP genes predominantly associated with hepatic metabolism were found to be absent or were expressed only in small amounts. Nevertheless, CYP2E1 and, in particular, CYP2D6 appeared to be noteworthy, as our rCYP analyses indicated that LTG can be metabolized by both enzymes. Furthermore, high affinity of LTG for CYP2D6 has been reported.^15^ This may indicate that even small amounts of the enzyme could contribute to the formation of metabolites in the skin.

Furthermore, again consistent with previous reports,^1,35,36^ our data revealed moderate to high expression of CYPs, which are associated with extrahepatic metabolism, including *CYP1A1*, *CYP1B1*, *CYP2J2*, and *CYP2S1*. These enzymes have previously been linked to the metabolism of drugs such as carvoxim or ebastine, where they are thought to be involved in the formation of highly reactive and sensitizing metabolites.^37,38^ Whether these enzymes could also be responsible for the metabolic processes of LTG remains to be seen.

With regard to phase II enzymes, our findings confirm the expression of several GST subtypes, in particular *GSTP1*, *GSTO1*, and *GSTK1*.^36,39,40^ High expression levels of GSTs are relevant, as they can detoxify reactive metabolites through enzymatic binding to GSH. At the same time, they are often recognized as macromolecules that form adducts with reactive metabolites. A growing number of studies report on these covalent drug–protein adducts, which may turn into haptens and thereby trigger cDHR.^9,41,42^

The levels of expression of UGTs and SULTs were generally low. We were unable to detect *UGT1A3* or *UGT1A4*, whereas *UGT2B7* was at least identified by qPCR. Several studies have discussed the role of UGT2B7 for LTG hepatic metabolism.^32,43,44^ Since UGT1A4 at least appears to be absent in keratinocytes, the role of UGT2B7 in LTG extrahepatic metabolism may be more relevant, especially for VPA interaction.^32^ VPA is thought to compete with the primary glucuronidation pathway of LTG, thereby increasing systemic LTG concentrations. This increase might lead to a shift to minor metabolic pathways, including CYP-mediated ones. In addition, it is known that reactive (toxic) metabolites of VPA are detoxified by conjugation with GSH, which, in turn, could significantly impair the detoxification of reactive LTG metabolites. A recent publication suggests that UGT2B10 could instead be involved.^45^

Moreover, three sulfotransferases, namely SULT1A1, SULT1A2, and SULT1C4, have recently been identified and hypothesized to play a role in LTG metabolism.^16^ Of these, SULT1A1 appears to be mainly expressed in the skin.^26,40^ However, no LTG-N2-sulfate could be observed in our subcellular assays, presumably due to its high reactivity and instability in water, even though it was detectable immediately after synthesis.

In terms of phase III enzymes, our sequencing data identified several ABC transporters and SLCs that have previously been described in keratinocytes and may also be linked to LTG.^4,46,47^ Among these are ABCC3 and ABCC4, both of which are known to transport LTG-N2-glucuronide in the liver.^22^

### Properties of a Possible Additional LTG Metabolite

In addition to previously reported LTG metabolites, such as the LTG-N2-oxide, the LTG-N2-glucuronide, and the GSH adduct,^22^ we identified another oxidative metabolite of LTG. While a compound with the same nominal mass and fragmentation pattern has been reported in plant metabolism studies,^48^ its presence in mammalian systems, particularly in the context of human LTG metabolism, has, to our knowledge, not been described.

LTG-OH was initially detected in RLM and eluted significantly earlier than LTG-N2-oxide, indicating increased polarity as CYP-mediated oxidation at the dichlorophenyl group has a more hydrophilic effect. This is further supported by characteristic fragment ions at *m*/*z* 236 (−Cl), 227 (−N_3_), and 188, which are expected for a hydroxylated phenyl-substituted triazine. While the fragment masses differ from those of LTG (*m*/*z*: + 16), the fragmentation remains structurally analogous, indicating preservation of the triazine core in contrast to the LTG-N2-oxide.^49^

To assess its relevance in human metabolism, the formation of LTG-OH was additionally tested in rCYP assays, where LTG-OH was mainly formed with rCYP2D6. Importantly, its formation coincided with the generation of both LTG-N2-oxide and the GSH conjugate. A reciprocal relationship was observed: CYPs that produced higher levels of the GSH adduct also generated more LTG-OH, whereas the level of LTG-N2-oxide formation was reduced. Conversely, when LTG-N2-oxide predominated, both LTG-OH and the GSH adduct were comparatively decreased. These results suggest that LTG-OH and the GSH adduct originate from a shared reactive epoxide intermediate with LTG-OH forming via spontaneous hydrolysis.

The analysis of deuterium-labeled LTG revealed the loss of one deuterium, consistent with an NIH shift and providing direct evidence of CYP-mediated aromatic oxidation.^50^ Nevertheless, the exact underlying oxidation mechanism (hydroxylation or epoxidation) is still unclear, as is the exact position of the hydroxyl group, which could be determined using ^1^H NMR. Beyond that, QM-MD simulations could provide deeper insights into the mechanistic steps involved in the formation of the LTG-OH metabolite, similar to what has been described previously for coumarin.^51^

Helpful information about the underlying mechanism could also be extrapolated from the structurally related compound 1,2-dichlorobenzene. This compound has long been known for its metabolism-dependent hepatotoxicity and is also oxidized to an arene oxide in rat and human liver microsomes, predominantly via CYP2E1.^52^ Similarly, the reactive metabolite is inactivated by glutathione conjugation but can also be converted to 2,3- or 3,4-dichlorophenol. It therefore seems at least reasonable that LTG undergoes analogous processes, despite the additional 2,4-triazine-3,5-diamine ring. Furthermore, the formation of an arene oxide has also been demonstrated for other aromatic antiepileptic drugs, e.g., carbamazepine or phenytoin.^53,54^

From a clinical perspective, there have already been attempts to detect LTG-N2-oxide in plasma samples, but with insufficient consistency.^55,56^ If LTG-OH levels correlate with the occurrence of skin rashes, then this metabolite could potentially serve as a biomarker for cDHR.

## Conclusion

This study revisits the basal expression of phase I, II, and III enzymes in keratinocytes. At the same time, it expands and confirms previous work by profiling all currently known LTG metabolites and potentially identifying one previously undescribed metabolite. Furthermore, we demonstrate the distinct effects of various CYP enzymes on LTG metabolism, raising the question of which of these enzymes may be primarily responsible for cDHR.

## Supplementary Material





## Data Availability

All data generated and analyzed during this study are included in the manuscript. The RNA sequencing data discussed in this publication have been deposited in NCBI’s Gene Expression Omnibus^57^ and are accessible through GEO Series accession number GSE324594 (https://www.ncbi.nlm.nih.gov/geo/query/acc.cgi?acc=GSE324594).

## Notes

Overview of considerations regarding the experimental approach (Scheme S1), identification of LTG-N2-sulfate using LC-MS without any purification (Figure S1), thin-layer chromatography of LTG, LTG-N2-oxide, and LTG-N2-sulfate (Figure S2), whole uncropped images of the original Western blots shown in Figure 1 (Figure S3), volcano plot of IFN-γ incubation after 2 h (A) and 6 h (B) (Figure S4), identification of LTG-N2-glucuronide in human liver microsomes (Figure S5), uncertain Identification of LTG-N2-glucuronide in keratinocytes after 24 h incubation (Figure S6), identification of LTG-N2-oxide in rat S9 fraction (Figure S7), identification of lamotrigine-glutathione in rat liver microsomes (Figure S8), additional Identification of LTG-OH and LTG-N2-oxide using Vanquish UHPLC coupled to an Orbitrap Exploris 480 (HR-MS) (Figure S9), formation of LTG-OH and LTG-N2-oxide by rCYPs (Figures S10–S14), simultaneous detection of LTG-GSH and LTG-OH by rCYP2D6 (Figure S15), formation of LTG-OH-d_2_ and LTG-N2-oxide by rCYP2D6 (Figure S16), primers used for reverse transcription-PCR analysis (Table S1) (PDF)
RNA expression in baseMean of all human xenobiotic-metabolizing enzymes and phase III (Table S2) (XLSX)

## Abbreviations

CYP: cytochrom P450 enzymes
cDHR: cutaneous drug hypersensitive reaction
ELISA: enzyme-linked immunosorbent assay
GSH: glutathione
HLC: human liver cytosol
HLM: human liver microsomes
RLM: rat liver microsomes
LTG: lamotrigine
ABC: ATP binding cassette
SLC: solute carrier
