# Supplementary material for: Drug-Metabolizing Enzymes in Human Keratinocytes and In Vitro Detection of Cytochrome P450-Mediated Phenolic Lamotrigine Metabolite
Source: Chem Res Toxicol. 2026 Mar 30;39(4):636–45. doi: 10.1021/acs.chemrestox.5c00500 (PMC13100949; doi:10.1021/acs.chemrestox.5c00500)
Supplement: Supplementary file 1 [file tx5c00500_si_001.pdf]

Supplementary Information

Drug-Metabolizing Enzymes in Human Keratinocytes and In Vitro Detection of Cytochrome P450-Mediated Phenolic Lamotrigine Metabolite

Philipp N. Deck,[†,⊥] Mareike Müller,[†,⊥] Andreas Glässner,[†] Matthias Vogel,[‡] Michael Steffens,[†] Caspar Heubach,[†] Guido Fechner,[§] Karl Becker,[ǁ] Günther Weindl,[⊥,#] and Bernhardt Sachs[*,†,#]

[†] Research Division, Federal Institute for Drugs and Medical Devices (BfArM), Bonn 53175, Germany

[‡] Pharmacopoeia and Standard Licensing Division, Federal Institute for Drugs and Medical Devices (BfArM), Bonn 53175, Germany

[§] Department of Urology and Pediatric Urology, University Hospital Bonn, Bonn 53127, Germany

[ǁ] Kinderchirurgische Gemeinschaftspraxis, Bonn 53113, Germany

[⊥] Pharmacology and Toxicology, Pharmaceutical Institute, University of Bonn, Bonn 53121, Germany

[#] *shared last authorship*

***Corresponding author**

Bernhardt Sachs – Research Division, Federal Institute for Drugs and Medical Devices (BfArM), Bonn 53175, Germany; Email: bernhardt.sachs@bfarm.de

Supplementary Information

Contents of Supplementary Information

Scheme S1. Overview of considerations regarding the experimental approach. S3

Figure S1. Identification of LTG-N2-sulfate using LC-MS without any purification. S4

Figure S2. Thin-layer chromatography of LTG, LTG-N2-oxide and LTG-N2-sulfate. S5

Figure S3. Whole uncropped images of the original Western blots shown in Figure 1. .... S6

Figure S4. Volcano plot of IFN-γ incubation after 2 hours (A) and 6 hours (B). S7

Figure S5. Identification of LTG-N2-glucuronide in human liver microsomes. S8

Figure S6. Uncertain identification of LTG-N2-glucuronide in keratinocytes after 24h incubation. S9

Figure S7. Identification of LTG-N2-oxide in rat S9 fraction. S10

Figure S8. Identification of lamotrigine-glutathione in rat liver microsomes S11

Figure S9. Additional identification of LTG-OH and LTG-N2-oxide using Vanquish UHPLC coupled to an Orbitrap Exploris 480 (HR-MS). S12

Figure S10. Formation of LTG-OH and LTG-N2-oxide by rCYP2A6 .... S13

Figure S11. Formation of LTG-OH and LTG-N2-oxide by rCYP2B6. .... S14

Figure S12. Formation of LTG-OH and LTG-N2-oxide by rCYP2D6 .... S15

Figure S13. Formation of LTG-OH and LTG-N2-oxide by rCYP2E1. .... S16

Figure S14. Formation of LTG-OH and LTG-N2-oxide by rCYP3A4 .... S17

Figure S15. Simultaneous detection of LTG-GSH and LTG-OH by rCYP2D6. .... S18

Figure S16. Formation of LTG-OH-d_2 and LTG-N2-oxide by rCYP2D6. .... S19

Table S1. Primers used for reverse transcription-PCR analysis. S20

Table S2. RNA expression in baseMean of all human xenobiotic-metabolizing enzymes and phase III transporters S21

Scheme S1. Overview of considerations regarding the experimental approach.

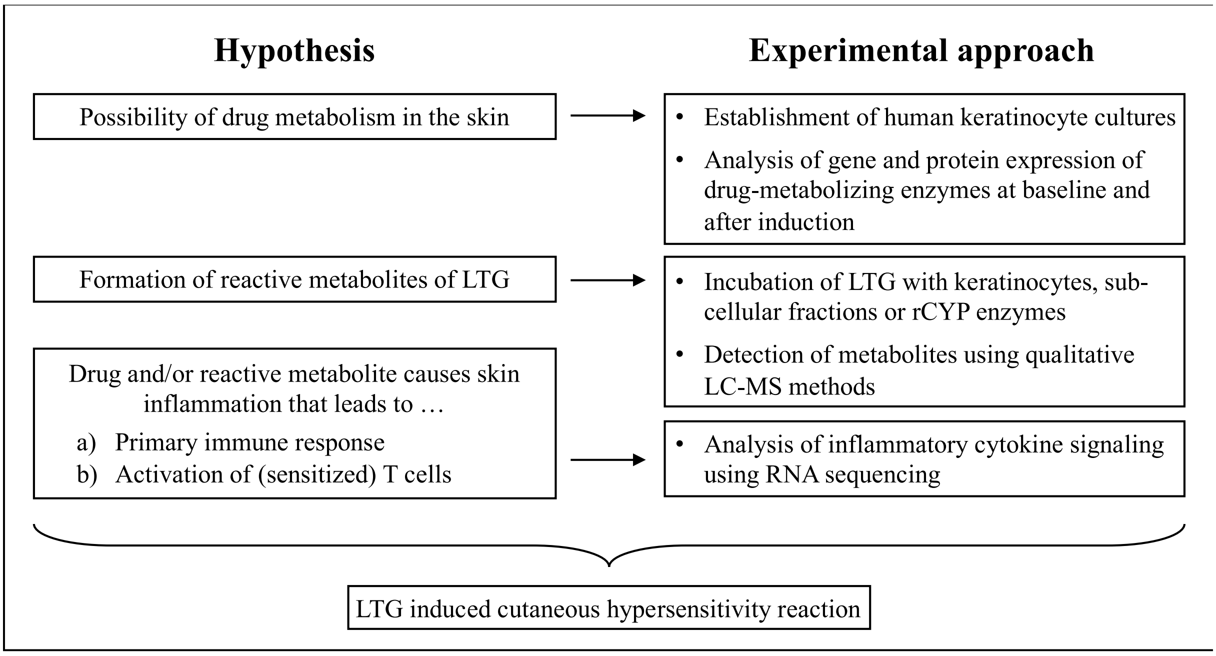

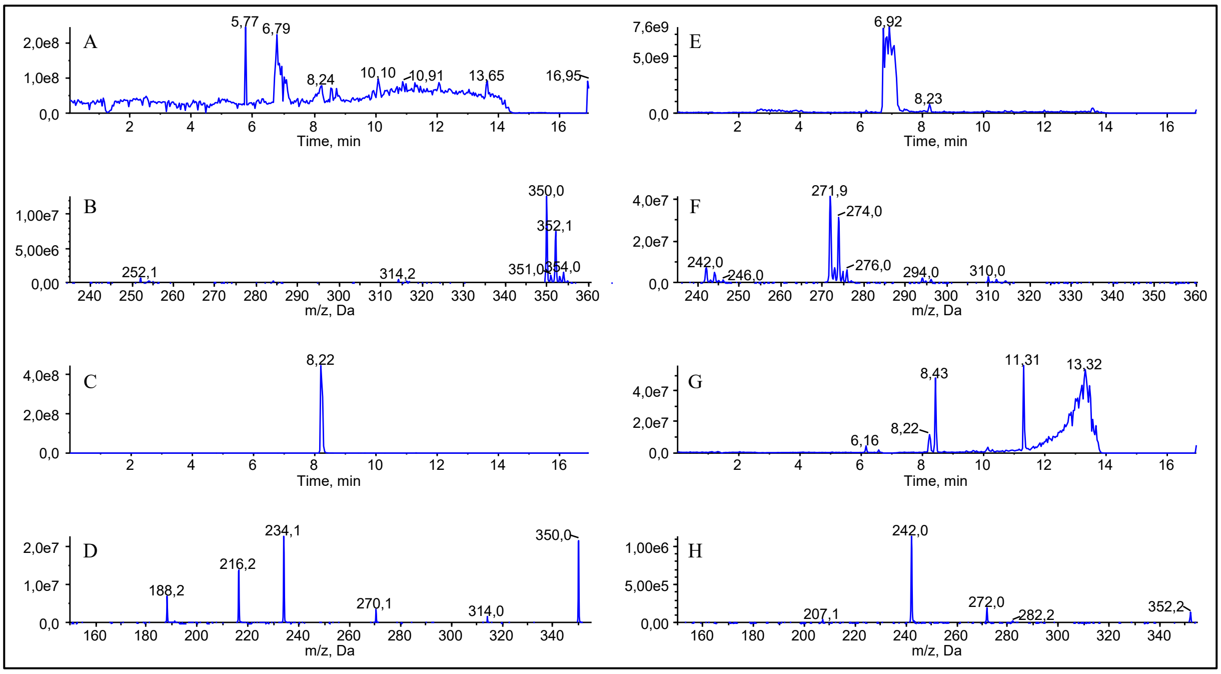

Figure S1. Identification of LTG-N2-sulfate using LC-MS without any purification.

Enhanced MS (EMS) chromatogram acquired in negative ESI mode and the corresponding mass spectrum at RT of 8.24 min (A-B). Enhanced Product Ion (EPI) chromatogram in negative ESI mode for m/z 350.0 and corresponding mass spectrum at RT of 8.22 min using a collision energy of -15 eV (C-D). EMS chromatogram acquired in positive ESI mode and the corresponding mass spectrum at RT of 8.23 min (E-F). EPI chromatogram in positive ESI mode for m/z 352.0 and corresponding mass spectrum at RT of 8.22 min using a collision energy of 15 eV (G-H). **UPLC**: 0.1% FA, B: ACN, Flow (μL/min): 350, 0.01 1% B, 0.50 1% B, 7.50 40% B, 12.50 100% B, 15.00 100% B 15.01 1% B, 17.00 1% B.

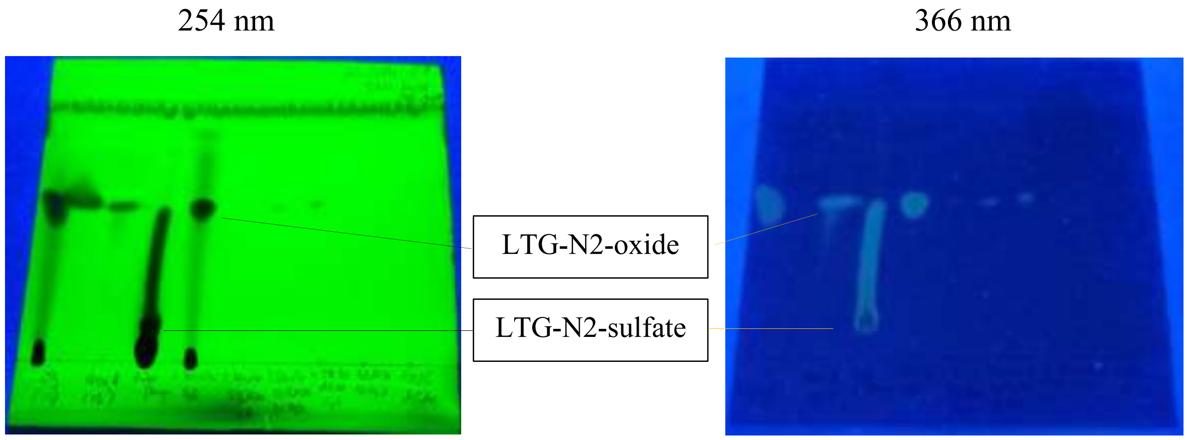

Figure S2. Thin-layer chromatography of LTG, LTG-N2-oxide and LTG-N2-sulfate.
TLC of the synthesized LTG-N2-sulfate using $CHCl_3$ / MeOH / H_2O / AcOH (65:25:5:1) as the mobile phase. LTG and LTG-N2-oxides were applied as references in lanes 1 and 2. Lane 3 contains the synthesis without purification attempts. Lanes 4 to 9 show fractions (ACN: 0, 5, 20, 50, 100%) of the SPE purification.

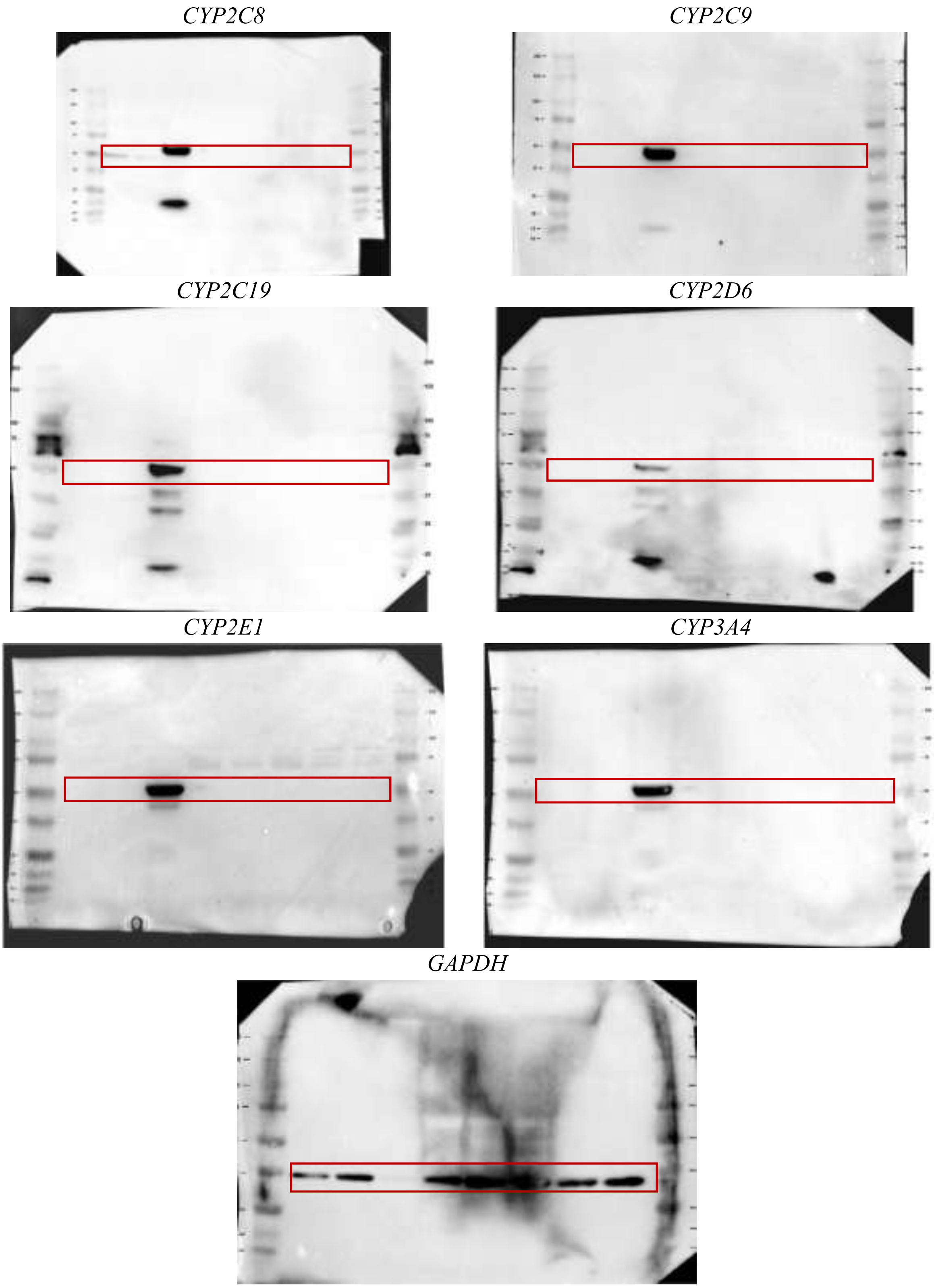

Figure S3. Whole uncropped images of the original Western blots shown in Figure 1.

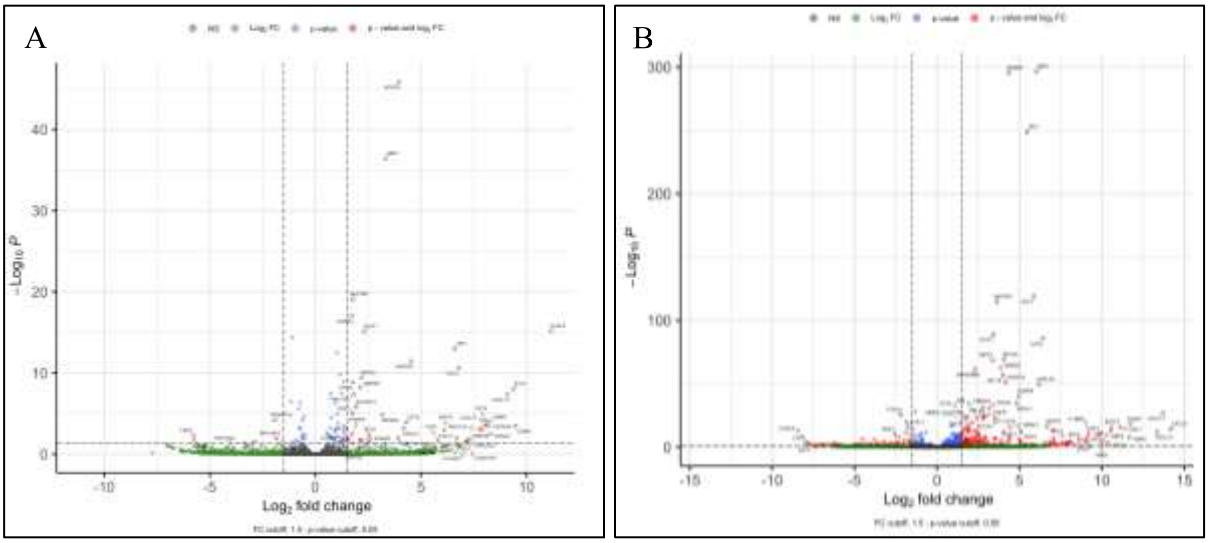

Figure S4. Volcano plot of IFN-γ incubation after 2 hours (A) and 6 hours (B).

Induction of several genes involved in the IFN-γ signaling pathway was detected after incubation of keratinocytes with IFN-γ. Significantly upregulated genes ($log_2FC > 1.5$, adjusted p value < 0.05) are highlighted in red, and downregulated genes ($log_2FC < -1.5$, adjusted $p < 0.05$) in blue. Non-significant changes are shown in gray.

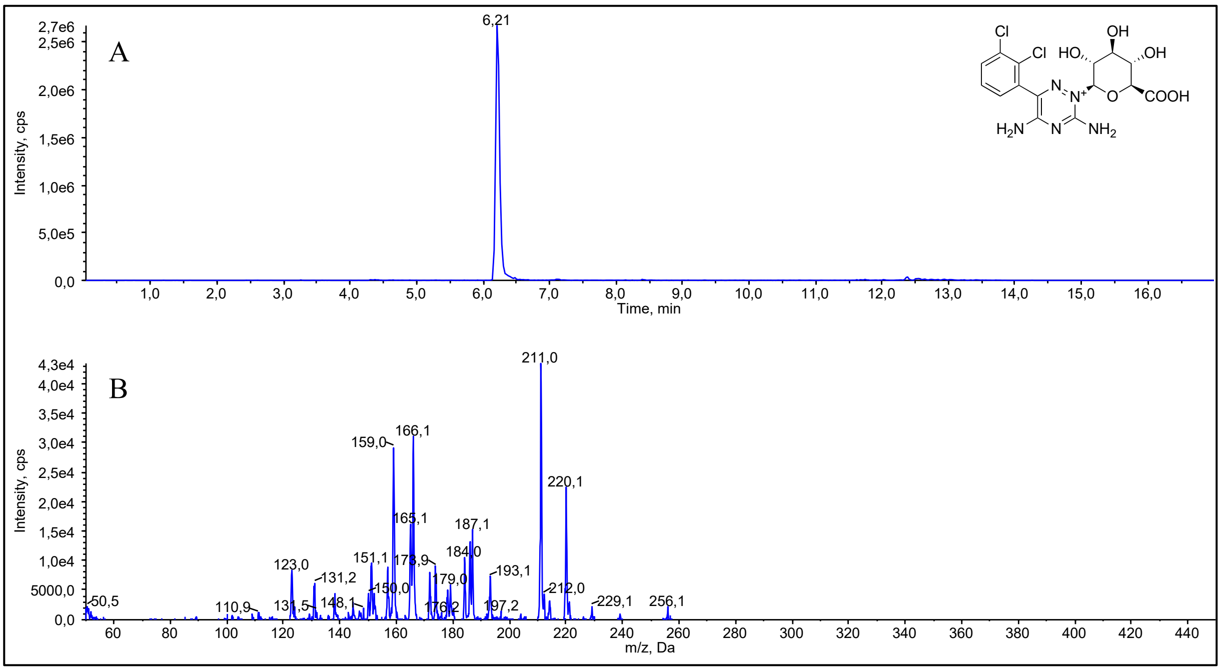

Figure S5. Identification of LTG-N2-glucuronide in human liver microsomes.

Representative LC-MS spectrum of lamotrigine-N2-glucuronide detected in HLM. The LC-MS^3 chromatogram and corresponding mass spectrum (A-B) shows a clear peak at 6.21 min corresponding to the molecular ion at m/z 432.0 → 256.0. The spectra were acquired in positive ESI mode. **MS^3**: CUR: 40, TEM: 450, GS1: 45, GS2: 20, IS: 5500, DP: 50, EP: 10, CE: 20, AF2: 0.10; **UPLC**: 0.1 % FA, B: ACN, Flow (µL/min): 350, 0.01 1% B, 9.00 40% B, 12.00 100% B, 15.00 50% B, 15.01 1% B, 17.00 1% B.

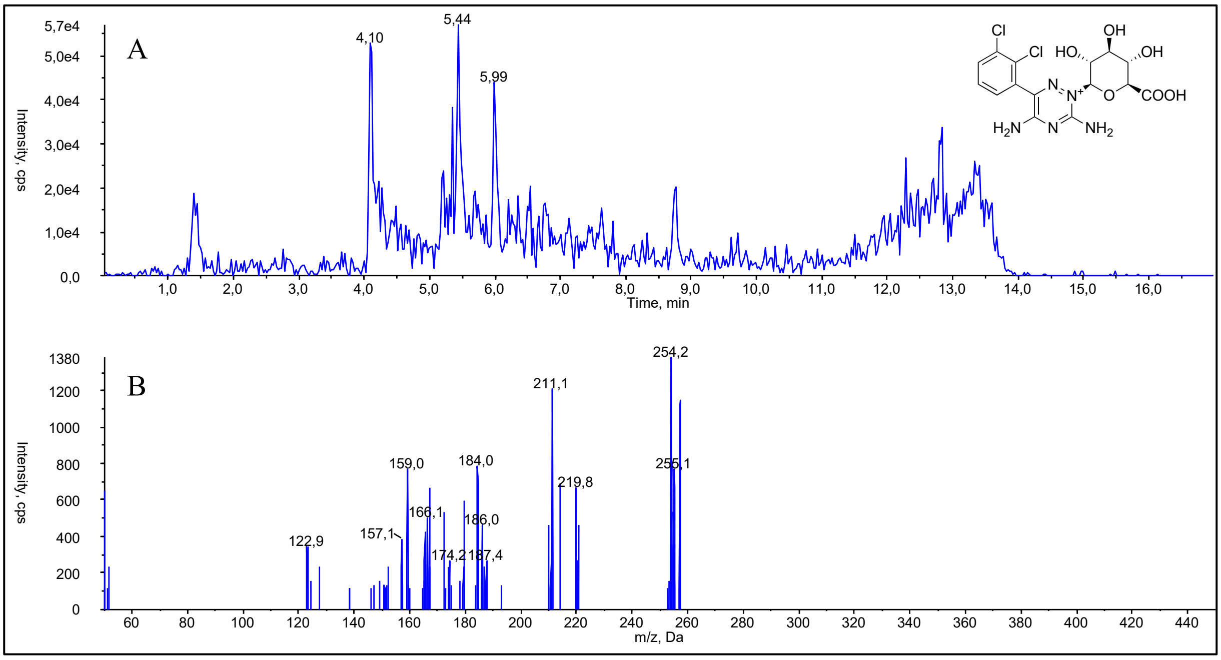

Figure S6. Uncertain identification of LTG-N2-glucuronide in keratinocytes after 24h incubation.

The LC-MS^3 chromatogram and corresponding mass spectrum (A-B) shows a peak at 5.99 min corresponding to the molecular ion at m/z 432.0 → 256.0. The spectra were acquired in positive ESI mode. **MS³**: CUR: 40, TEM: 450, GS1: 45, GS2: 20, IS: 5500, DP: 50, EP: 10, CE: 20, AF2: 0.10; **UPLC**: 0.1 % FA, B: ACN, Flow (μL/min): 350, 0.01 1% B, 9.00 40% B, 12.00 100% B, 15.00 50% B, 15.01 1% B, 17.00 1% B.

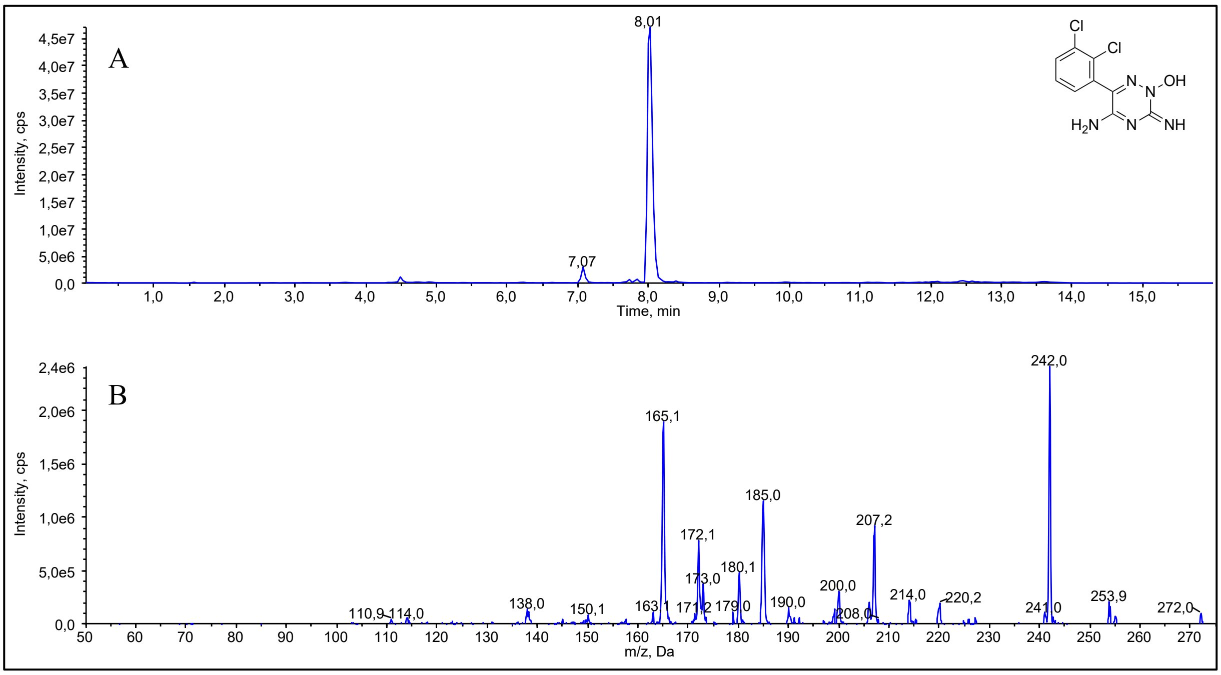

Figure S7. Identification of LTG-N2-oxide in rat S9 fraction.

Representative LC-MS spectrum of lamotrigine-N2-oxide detected in rS9. The EPI chromatogram in positive ESI mode for m/z 272.0 and corresponding mass spectrum at RT of 8.01 min using a collision energy of 30 eV (A-B). **MS^2**: CUR: 40, TEM: 450, GS1: 45, GS2: 20, IS: 5500, DP: 70, EP: 10, CE: 30; **UPLC**: 0.1% FA, B: ACN, Flow (µL/min): 350, 0.01 1% B, 9.00 40% B, 12.00 100% B, 15.00 50% B, 15.01 1% B, 17.00 1% B.

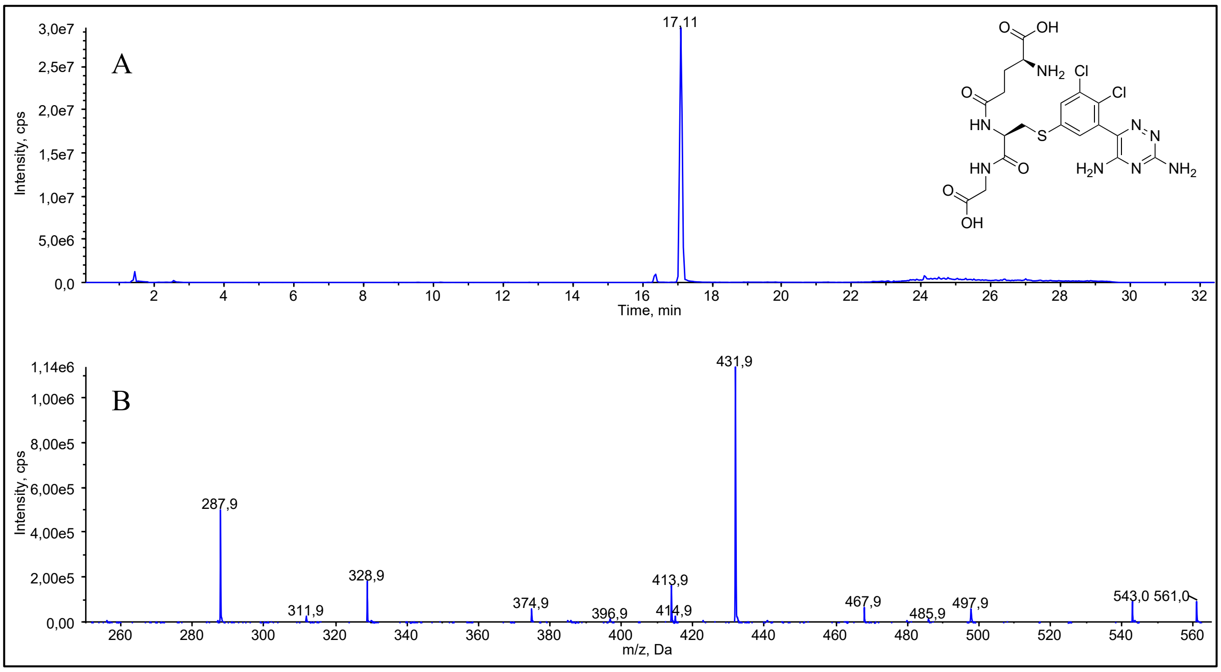

Figure S8. Identification of lamotrigine-glutathione in rat liver microsomes

Representative LC-MS spectrum of lamotrigine-glutathione detected in RLM. The EPI chromatogram in positive ESI mode for m/z 561.0 and corresponding mass spectrum at RT of 17.11 min using a collision energy of 25 eV (A-B). **MS^2**: CUR: 40, TEM: 450, GS1: 45, GS2: 20, IS: 5500, DP: 100, EP: 10, CE: 25; **UPLC**: 5 mM NH_4Ac, B: ACN, Flow (μL/min): 350, 0.01 1% B, 20.00 20% B, 22.50 50% B, 27.50 50% B, 27.52 1% B, 32.50 1% B.

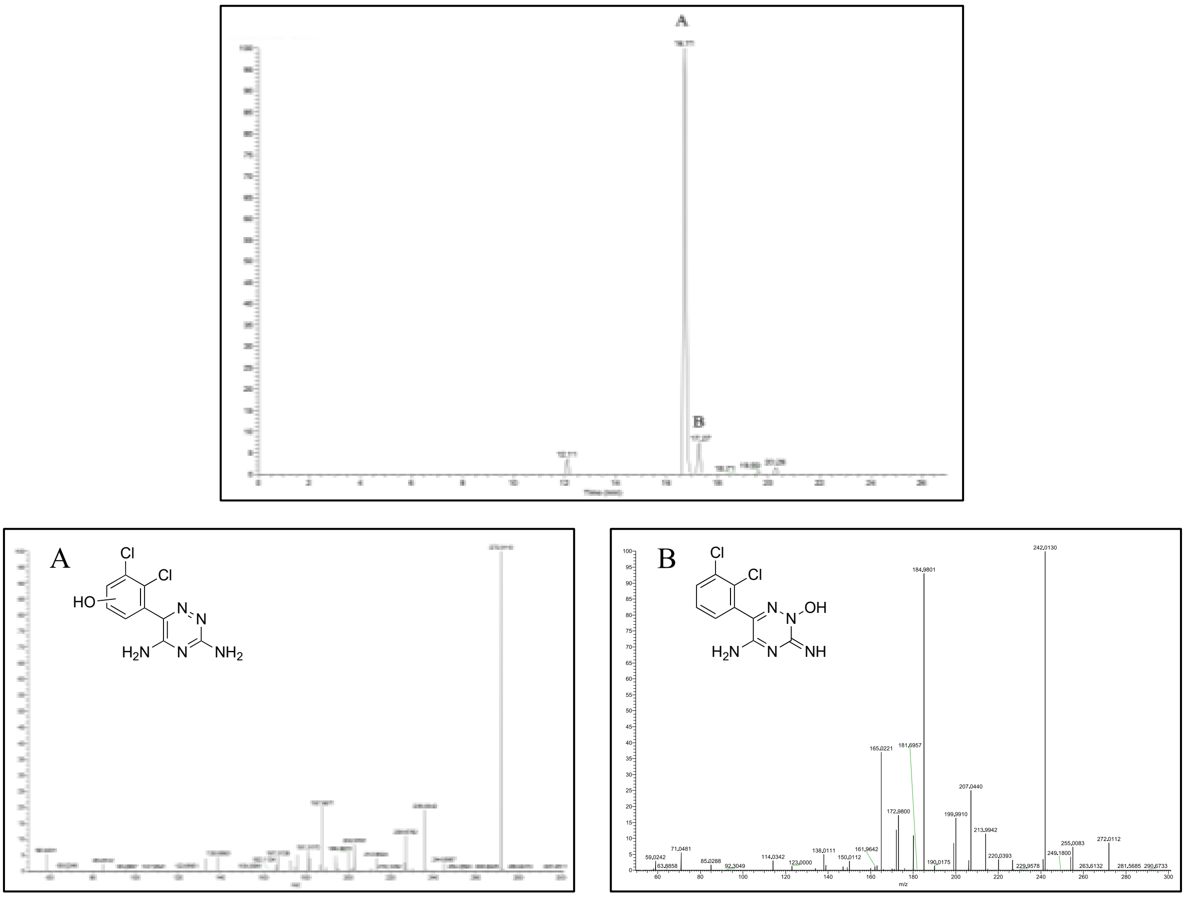

Figure S9. Additional identification of LTG-OH and LTG-N2-oxide using Vanquish UHPLC coupled to an Orbitrap Exploris 480 (HR-MS).

Full-MS chromatogram (top) and ESI-MS/MS spectrum (bottom) of rCYP2D6 assay. RT 16.71 (A) RT 17.27 (B). Settings: Spray Voltage: 3500 V, Ion Transfer Tube: 350 °C; **Full Scan** Resolution (FWHM at *m*/*z* 200): 60,000, Scan Range (*m*/*z*): 100-600; **tMS2** Resolution (FWHM at *m*/*z* 200): 45,000, Isolation Window (*m*/*z*): 1.4; Collision Energy Mode: Stepped, HCD Collision Energy (%): 15, 30, 45, 60; **UPLC**: 5 mM NH_4Ac + 0.1 % FA, B: ACN, Flow (µL/min): 350, 0.00 0% B, 20.00 20% B, 22.50 50% B, 25.00 50% B, 25.01 0% B, 27.00 0% B.

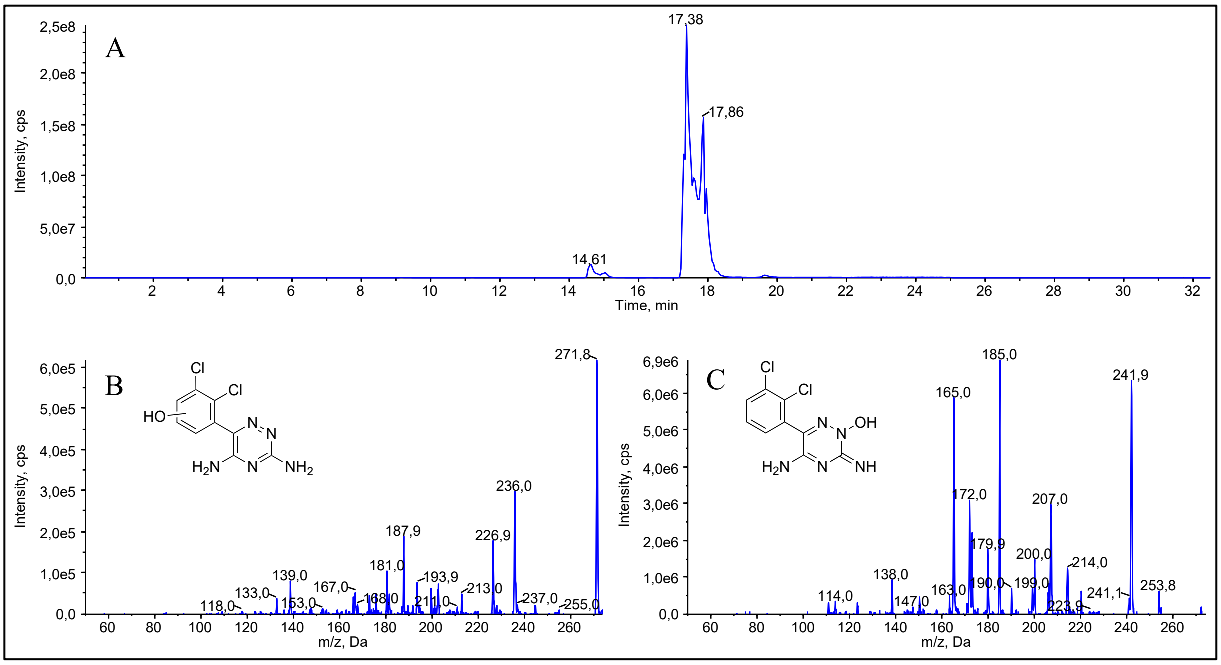

Figure S10. Formation of LTG-OH and LTG-N2-oxide by rCYP2A6.

Representative LC-MS spectrum for the rCYP2A6 assay. The EPI chromatogram in positive ESI mode for m/z 272.0 (A) and corresponding mass spectrum at RT of 14.61 min and 17.38 min using a collision energy of 35 eV (B-C). **MS^2**: CUR: 20, TEM: 0, GS1: 45, GS2: 20, IS: 5500, DP: 100, EP: 10, CE: 35; **UPLC**: 5 mM NH_4Ac, B: ACN, Flow (µL/min): 350, 0.01 1% B, 20.00 20% B, 22.50 50% B, 27.50 50% B, 27.52 1% B, 32.50 1% B.

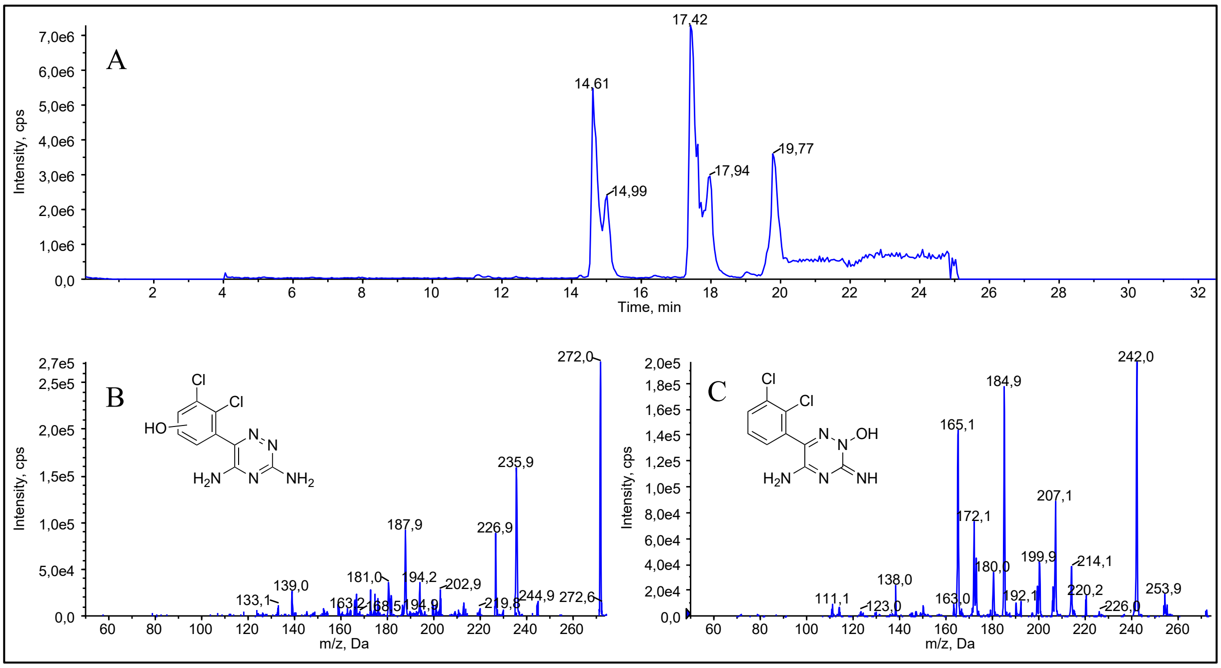

Figure S11. Formation of LTG-OH and LTG-N2-oxide by rCYP2B6.

Representative LC-MS spectrum for the rCYP2B6 assay. The EPI chromatogram in positive ESI mode for m/z 272.0 (A) and corresponding mass spectrum at RT of 14.61 min and 17.42 min using a collision energy of 35 eV (B-C). **MS^2**: CUR: 20, TEM: 0, GS1: 45, GS2: 20, IS: 5500, DP: 100, EP: 10, CE: 35; **UPLC**: 5 mM NH_4Ac, B: ACN, Flow (µL/min): 350, 0.01 1% B, 20.00 20% B, 22.50 50% B, 27.50 50% B, 27.52 1% B, 32.50 1% B.

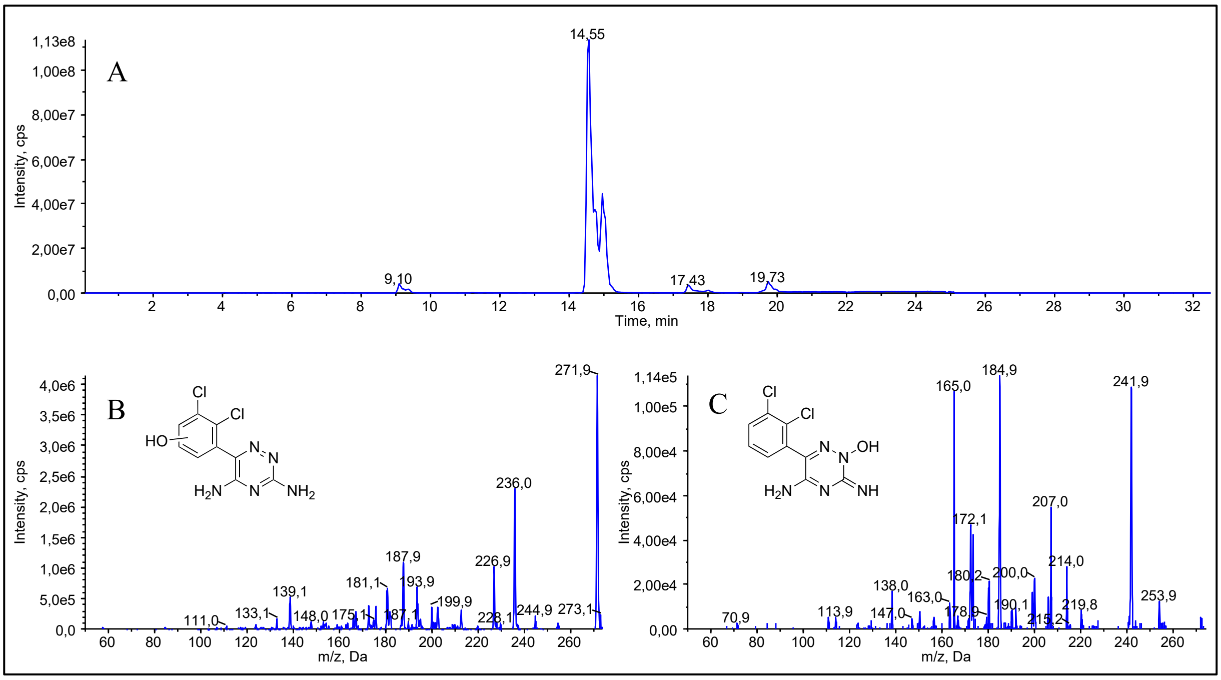

Figure S12. Formation of LTG-OH and LTG-N2-oxide by rCYP2D6.

Representative LC-MS spectrum for the rCYP2D6 assay. The EPI chromatogram in positive ESI mode for m/z 272.0 (A) and corresponding mass spectrum at RT of 14.55 min and 17.43 min using a collision energy of 35 eV (B-C). **MS^2**: CUR: 20, TEM: 0, GS1: 45, GS2: 20, IS: 5500, DP: 100, EP: 10, CE: 35; **UPLC**: 5 mM NH_4Ac, B: ACN, Flow (µL/min): 350, 0.01 1% B, 20.00 20% B, 22.50 50% B, 27.50 50% B, 27.52 1% B, 32.50 1% B.

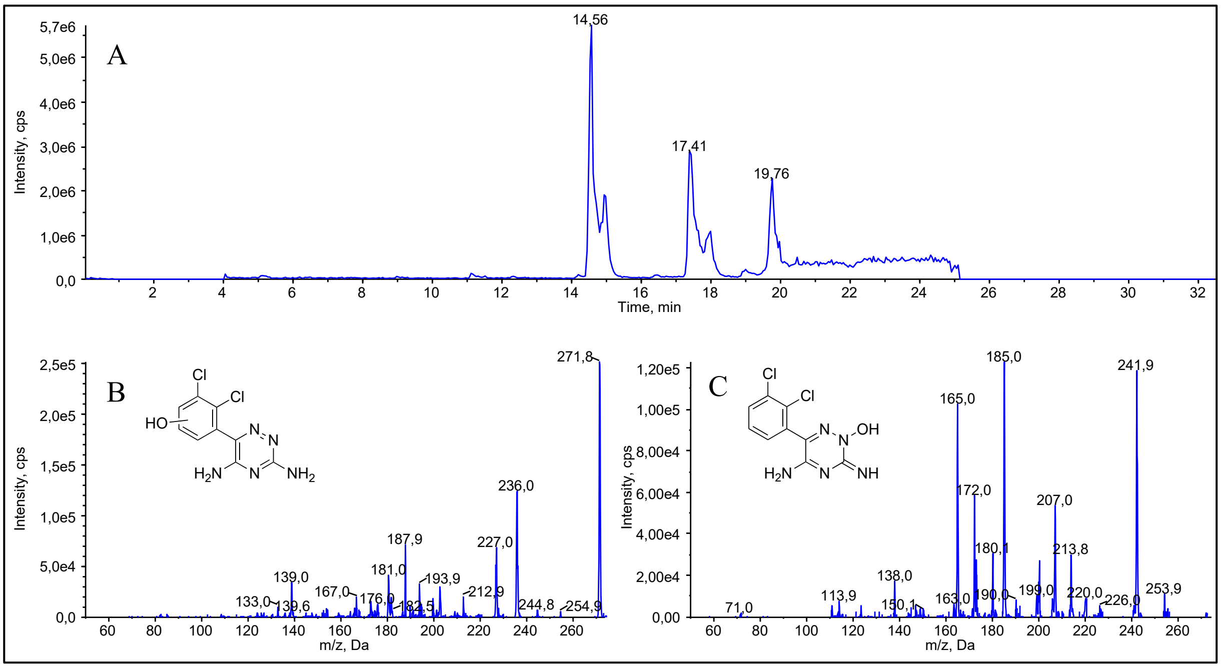

Figure S13. Formation of LTG-OH and LTG-N2-oxide by rCYP2E1.

Representative LC-MS spectrum for the rCYP2E1 assay. The EPI chromatogram in positive ESI mode for m/z 272.0 (A) and corresponding mass spectrum at RT of 14.56 min and 17.41 min using a collision energy of 35 eV (B-C). **MS^2**: CUR: 20, TEM: 0, GS1: 45, GS2: 20, IS: 5500, DP: 100, EP: 10, CE: 35; **UPLC**: 5 mM NH_4Ac, B: ACN, Flow (µL/min): 350, 0.01 1% B, 20.00 20% B, 22.50 50% B, 27.50 50% B, 27.52 1% B, 32.50 1% B.

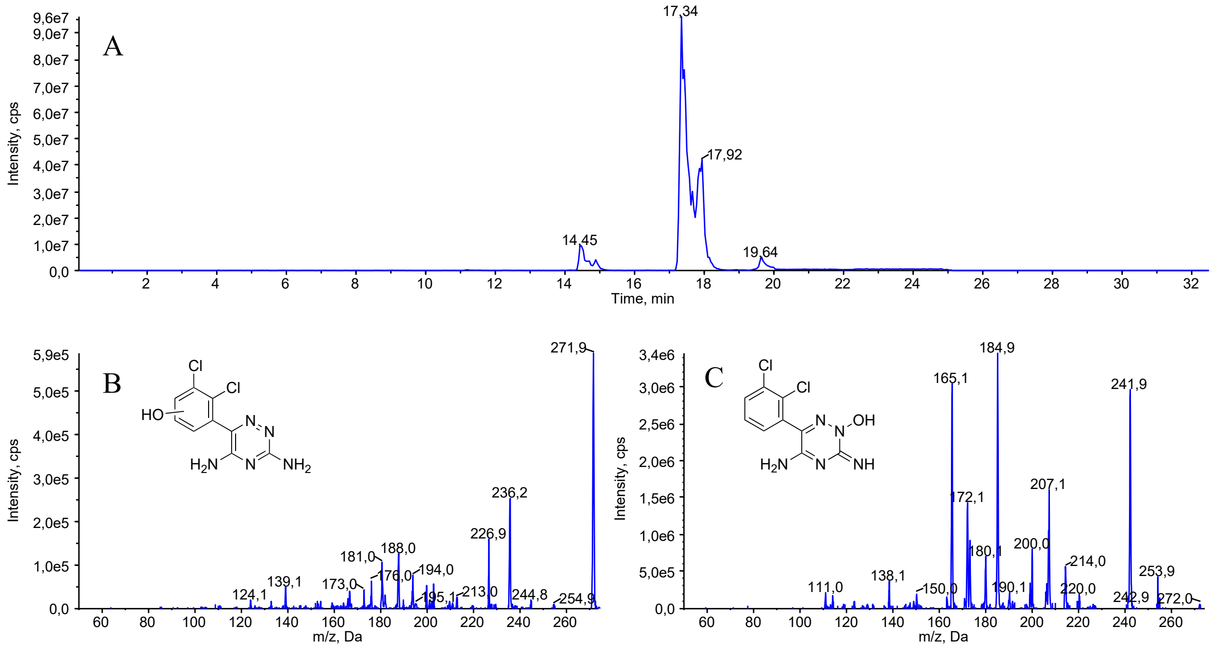

Figure S14. Formation of LTG-OH and LTG-N2-oxide by rCYP3A4.

Representative LC-MS spectrum for the rCYP3A4 assay. The EPI chromatogram in positive ESI mode for m/z 272.0 (A) and corresponding mass spectrum at RT of 14.56 min and 17.41 min using a collision energy of 35 eV (B-C). **MS²**: CUR: 20, TEM: 0, GS1: 45, GS2: 20, IS: 5500, DP: 100, EP: 10, CE: 35; **UPLC**: 5 mM NH_4Ac, B: ACN, Flow (µL/min): 350, 0.01 1% B, 20.00 20% B, 22.50 50% B, 27.50 50% B, 27.52 1% B, 32.50 1% B.

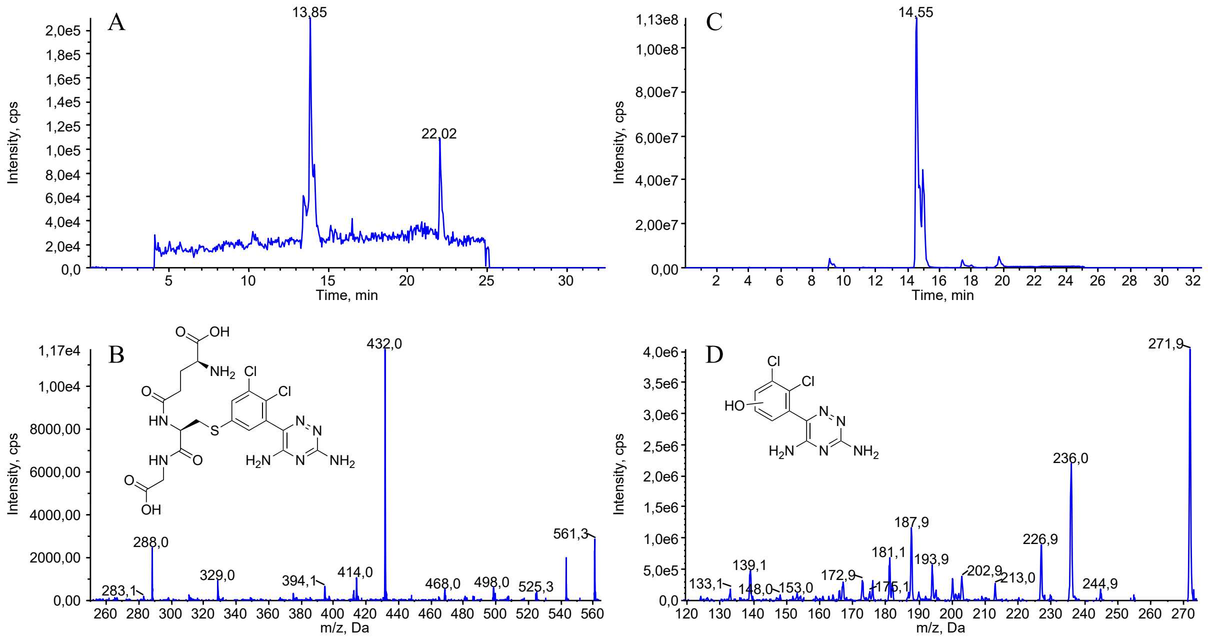

Figure S15. Simultaneous detection of LTG-GSH and LTG-OH by rCYP2D6.

Representative LC-MS spectrum for the rCYP2D6 assay. The EPI chromatogram in positive ESI mode for m/z 561.0 (A) and corresponding mass spectrum at RT of 13.85 min using a collision energy of 25 eV (B). The EPI chromatogram in positive ESI mode for m/z 272.0 (C) and corresponding mass spectrum at RT of 14.55 min using a collision energy of 35 eV (D). **MS^2**: CUR: 20, TEM: 0, GS1: 45, GS2: 20, IS: 5500, DP: 100, EP: 10, CE: 25; **UPLC**: 5 mM NH_4Ac, B: ACN, Flow (µL/min): 350, 0.01 1% B, 20.00 20% B, 22.50 50% B, 27.50 50% B, 27.52 1% B, 32.50 1% B.

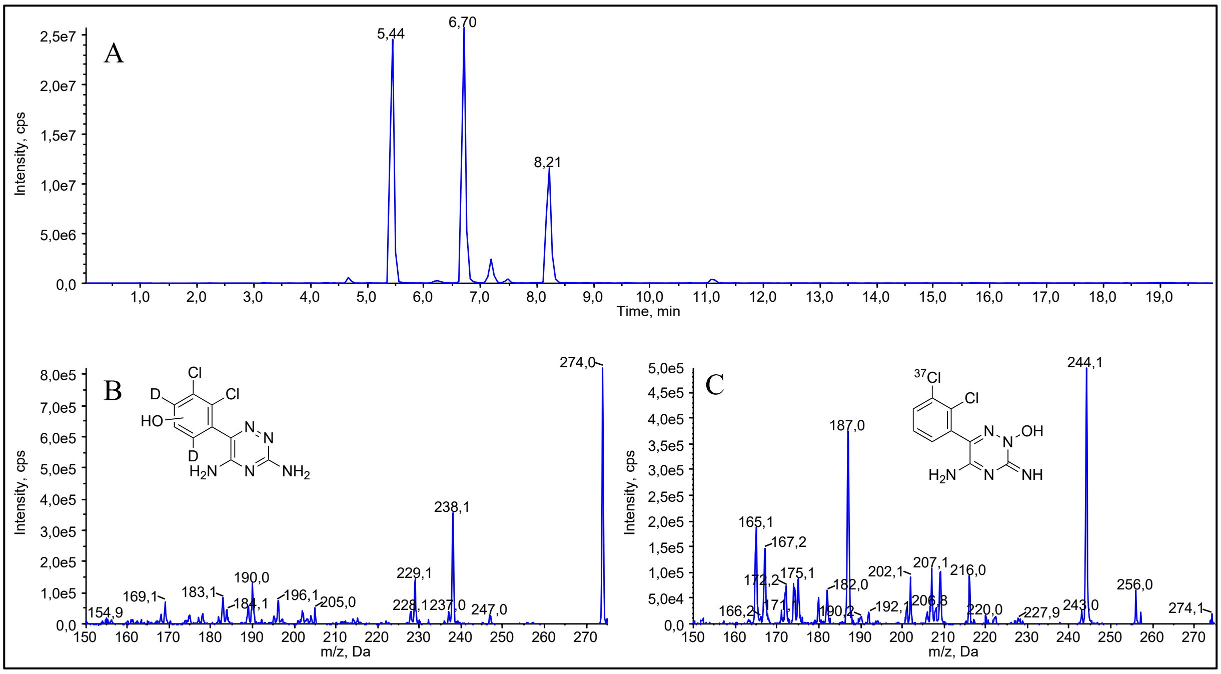

Figure S16. Formation of LTG-OH-d_2 and LTG-N2-oxide by rCYP2D6.

Representative LC-MS spectrum for the rCYP2D6 assay. The EPI chromatogram in positive ESI mode for m/z 272.0 (A) and corresponding mass spectrum at RT of 5.44 min and 6.70 min using a collision energy of 35 eV (B-C). **MS^2**: CUR: 20, TEM: 0, GS1: 45, GS2: 20, IS: 5500, DP: 100, EP: 10, CE: 35; **UPLC**: 0.1% FA, B: ACN, Flow (μL/min): 350, 0.01 1% B, 2.00 20% B, 15.00 50% B, 17.00 50% B, 17.01 1% B, 20.00 1% B.

Table S1. Primers used for reverse transcription-PCR analysis.
CYP2B6 (Hs_CYP2B6_1_SG, QT00000910), KRT14 (Hs_KRT14_1_SG, QT00052283) and LOR (Hs_LOR_1_SG, QT00209307) were QuantiTect Primer. Following KiCqStart-Primer were used:

Gene	Forward primer	Reverse primer
CYP1B1	CCTCTTCACCAGGTATCC	ATGAGGAATAGTGACAGGC
CYP2A6	TAGAGGGAAGAGAAGAAACAG	TTTGCTCAGGAAATAAGAGC
CYP2C8	CCAAATATCTTTGACCCTGG	AAATTCGTTTTCCTGCTGAG
CYP2C9	GGAATTGTTTTCAGCAATGG	CTTGGTTTTTCTCAACTCCTC
CYP2C19	CATGGATATGAAGTGGTGAAG	TCCATTGCTGAAAACGATTC
CYP2D6	CTGGCAGAGATGGAGAAG	TGTAGGATCATGAGCAGG
CYP2E1	TAATGAGAACTTCCACCTACTC	TCACCCTTTCAGACACATAC
CYP2J2	AGCAATAAAAGAGGAGAACG	CTCCAAGTATGTGACTTCATC
CYP3A4	AGTCTTTCCATTCCTCATCC	TGCTTTTGTGTATCTTCGAG
SULT1A1	CTTCTATGAAGACATGAAGGAG	TGTAGTTGGTCATAGGGTTC
SULT1A2	CCTGTTCTCTACCTCTTCTATG	CTTCTTCATCTCCTTGAACG
SULT1C4	GATCCTGAAAACACATCTTCC	ATACTCTTCCCATGTTCCTG
UGT1A1	GTGACTTTGTGAAGGATTACC	TCCTGGGATAGTGGATTTTG
UGT1A4	CTTTTTCAGAGAGAGGTGTC	ATTCCTGAGATAGTGGCTTC
UGT2B7	ACGTATGGCTTATTCGAAAC	CATGTTACTGACCATTGACC
PTGS1	GTTCTGGGAGTTTGTCAATG	GAGTGTAATAGCTCACGTTG
PTGS2	AAGCAGGCTAATACTGATAGG	TGTTGAAAAGTAGTTCTGGG
COMT	ACAAGAAAGGCAAGATCG	TGATCTCGATGGTGATGAG
GAPDH	CTTTTGCGTCGCCAG	TTGATGGCAACAATATCCAC

Table S2. RNA expression in baseMean of all human xenobiotic-metabolizing enzymes and phase III transporters

Further information can be found in Supplementary Excel Sheet ‘Supplementary_RNA-Seq’.
